# Targeted Repair of p47-CGD in iPSCs by CRISPR/Cas9: Functional Correction without Cleavage in the Highly Homologous Pseudogenes

**DOI:** 10.1016/j.stemcr.2019.08.008

**Published:** 2019-09-19

**Authors:** Denise Klatt, Erica Cheng, Friederike Philipp, Anton Selich, Julia Dahlke, Reinhold E. Schmidt, Juliane W. Schott, Hildegard Büning, Dirk Hoffmann, Adrian J. Thrasher, Axel Schambach

**Affiliations:** 1 Institute of Experimental Hematology, Hannover Medical School, Carl-Neuberg-Strasse 1, 30625 Hannover, Germany; 2 REBIRTH Cluster of Excellence, Hannover Medical School, 30625 Hannover, Germany; 3 Fraunhofer Institute for Toxicology and Experimental Medicine, 30625 Hannover, Germany; 4 Department of Immunology and Rheumatology, Hannover Medical School, 30625 Hannover, Germany; 5 Infection, Immunity and Inflammation Program, Molecular and Cellular Immunology Section, UCL Great Ormond Street Institute of Child Health, University College London, London WC1N 1EH, UK; 6 Great Ormond Street Hospital NHS Foundation Trust, London WC1N 1EH, UK; 7 Division of Hematology/Oncology, Boston Children's Hospital, Harvard Medical School, Boston, MA 02115, USA

**Keywords:** chronic granulomatous disease (CGD), NADPH oxidase, p47phox, NCF1, CRISPR/Cas9, human induced pluripotent stem cells, gene editing, pseudogenes

## Abstract

Mutations in the NADPH oxidase, which is crucial for the respiratory burst in phagocytes, result in chronic granulomatous disease (CGD). The only curative treatment option for CGD patients, who suffer from severe infections, is allogeneic bone marrow transplantation. Over 90% of patients with mutations in the p47^phox^ subunit of the oxidase complex carry the deletion c.75_76delGT (ΔGT). This frequent mutation most likely originates via gene conversion from one of the two pseudogenes *NCF1B* or *NCF1C*, which are highly homologous to *NCF1* (encodes p47^phox^) but carry the ΔGT mutation. We applied CRISPR/Cas9 to generate patient-like p47-ΔGT iPSCs for disease modeling. To avoid unpredictable chromosomal rearrangements by CRISPR/Cas9-mediated cleavage in the pseudogenes, we developed a gene-correction approach to specifically target *NCF1* but leave the pseudogenes intact. Functional assays revealed restored NADPH oxidase activity and killing of bacteria in corrected phagocytes as well as the specificity of this approach.

## Highlights

• CRISPR/Cas9-mediated disease modeling of p47-CGD in iPSCs mimics pathogenesis
• Targeted repair allows correction of p47-CGD without cleavage in the pseudogenes
• Corrected iPSC-derived granulocytes have a restored NADPH oxidase function
• Corrected iPSC-derived macrophages phagocytose and kill bacteria

## Graphical Abstract

**Figure:**
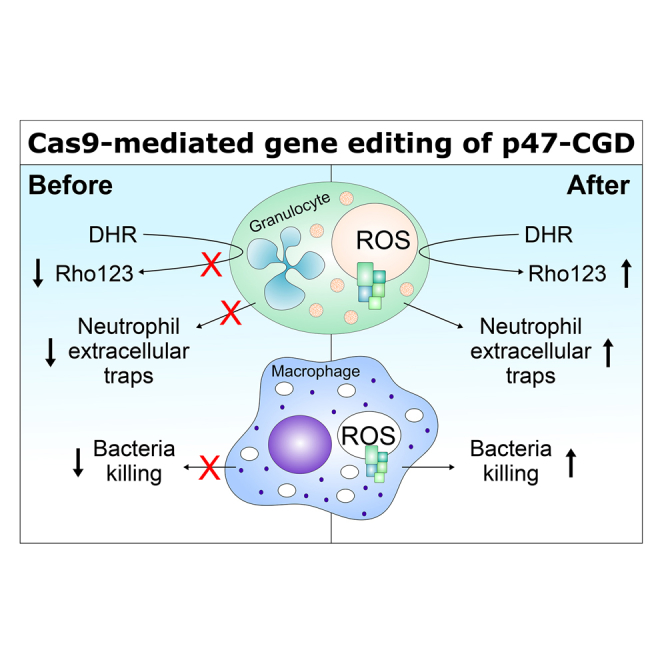

## Teaser

Klatt, Schambach and colleagues generated patient-like p47-ΔGT iPSCs for disease modeling of chronic granulomatous disease and subsequent gene correction of *NCF1* (encodes p47^phox^) using CRISPR/Cas9. Their strategy avoided cleavage of the highly homologous *NCF1* pseudogenes, which might have a functional role. Corrected granulocytes and macrophages demonstrated restored NADPH oxidase activity, formation of neutrophil extracellular traps, and killing of phagocytosed bacteria.

## Introduction

Loss-of-function mutations in the NADPH oxidase cause chronic granulomatous disease (CGD), a rare primary immunodeficiency characterized by defective phagocytes unable to regulate efficient killing of engulfed pathogens by granule proteases (Reeves et al., 2002, Roos, 1994). Thus, patients suffer from recurrent bacterial and fungal infections and rely on lifelong antibiotic and antifungal prophylaxis (Seger, 2008). Despite improved control of the disease, many patients still develop severe infections that are often refractory to conventional therapy (Holland, 2010). To combat these infections, patients receive cell-based therapies, such as granulocyte transfusions or allogenic hematopoietic stem cell transplantation (HSCT) (Güngör et al., 2014, Marciano et al., 2017a). The latter is the only curative treatment option. Early efforts to treat CGD by gammaretroviral gene therapy were complicated by mutagenesis (Ott et al., 2006). More recent studies using chimeric regulatory sequences in a lentiviral vector platform are showing significant promise, although ultimately a precise gene-editing approach is an attractive option.

While the disease-causing mutations are very heterogeneous for most NADPH oxidase subunits, over 90% of patients with a mutation in the p47^phox^ subunit (encoded by *NCF1*), carry the deletion c.75_76delGT (ΔGT) in the 5′ region of exon 2 (Roos et al., 2010). The high prevalence of this mutation (25% of overall CGD patients) most likely originates via gene conversion from one of the two pseudogenes *NCF1B* and *NCF1C*, which also harbor the ΔGT mutation, have a sequence homology of over 99% compared with *NCF1*, and are in close proximity to *NCF1* (Roesler et al., 2000). The functional role of the pseudogenes is as yet not well studied. Xu et al. (2015) demonstrated that overexpression of certain pseudogene transcripts resulted in downregulation of superoxide production in endothelial cells, indicating a functional role. Moreover, Merling et al. (2017) showed that correction of the ΔGT mutation in the pseudogenes using zinc-finger nucleases restored protein expression and resulted in functional NADPH oxidase activity. However, directly targeting the ΔGT mutation carries the risk of chromosomal aberrations. Every designer nuclease, even the highly site-specific CRISPR/Cas9 system, will potentially introduce two DNA double-strand breaks (DSB) into *NCF1* and into each pseudogene due to their sequence homology. Consequently, the repair of up to six DSBs can result in larger deletions, inversions, or translocations, which could potentially cause severe, unwanted alterations in the genome.

Human induced pluripotent stem cells (iPSCs) are used in many research fields as disease models to develop novel treatment strategies. However, patient-derived cells, such as peripheral blood mononucleated cells (PBMCs) or skin fibroblasts, are needed for reprogramming, and primary material can be difficult to obtain when studying rare diseases. Here, we used CRISPR/Cas9 to generate patient-like p47-ΔGT iPSCs for disease modeling and to develop our gene-correction approach. In contrast to retroviral vectors, the CRISPR/Cas9 system can be applied transiently and allows direct correction of the mutated gene with the advantage that the expression is driven by the endogenous promoter while the original genome architecture remains unaltered. We demonstrate proof of principle that the insertion of a minigene into intron 1 of *NCF1* can correct p47^phox^ deficiency. To specifically target *NCF1*, we chose a position in intron 1 that differs from the pseudogene sequence by an additional three base pairs; thus, the pseudogenes were not targeted by Cas9 and remained intact. Myeloid differentiation of corrected iPSCs revealed restored p47^phox^ expression and functional NADPH oxidase activity measured by reactive oxygen species (ROS) production, formation of neutrophil extracellular traps (NETs), and killing of bacteria.

## Results

### Generation of Patient-like p47-ΔGT iPSCs Using CRISPR/Cas9

**Figure 1:**
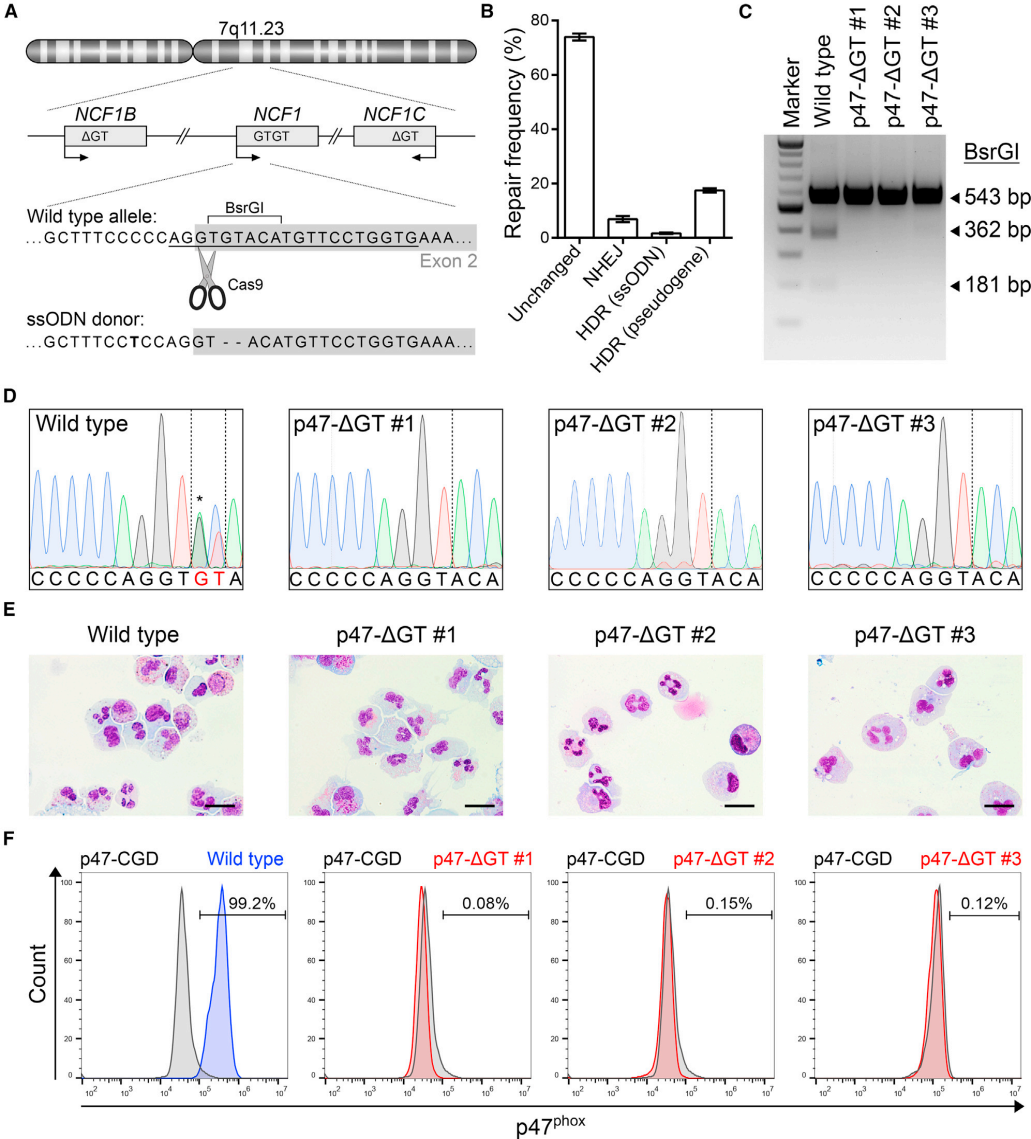
Generation of Patient-like p47-ΔGT iPSCs Using CRISPR/Cas9 (A) Schematic of *NCF1* and its pseudogenes *NCF1B/C* located on chromosome 7. The sgRNA target sequence (underlined) is shown on the wild type allele in the 5′ region of exon 2 (gray box). The donor template (ssODN) carries the ΔGT mutation and a silent point mutation (bold). (B) Frequency of repaired alleles by non-homologous end-joining (NHEJ) or homology-directed repair (HDR) after Cas9-mediated cleavage. The frequency was determined in sorted bulk populations by next-generation sequencing. The name in parentheses indicates the donor template used (n = 3, mean ± SD). (C) PCR amplification and BsrGI digest to discriminate correctly modified iPSC clones (BsrGI site removed) from wild type (BsrGI site present). (D) Sanger sequencing results of wild type iPSCs and p47-ΔGT clones. The wild type sequence shows overlaid sequences of *NCF1* and the pseudogenes due to co-amplification via PCR (asterisk). (E) Cell morphology of differentiated iPSC-derived granulocytes for wild type and p47-ΔGT cells after Pappenheim staining. Scale bars, 20 μm. (F) Intracellular staining followed by flow cytometry to detect p47^phox^ expression in wild type and p47-ΔGT granulocytes (gated on p47-CGD granulocytes).

In former CGD iPSC disease models, patient cells were reprogrammed and the disease phenotype was monitored in differentiated hematopoietic cells in comparison with iPSC-derived cells from healthy individuals. However, donor-specific gene expression and genetic variations of iPSC lines may affect the developmental potential (Mills et al., 2013). To investigate the CGD disease phenotype in the same, and therefore comparable, genetic background, we introduced the p47-ΔGT mutation into a well-characterized iPSC line derived from CD34^+^ blood cells (Ackermann et al., 2014). On-target activity of the specifically designed single guide RNA (sgRNA) p47.ex2 as well as off-target activity in the highly homologous pseudogenes was assessed in an HT1080 reporter cell assay using lentiviral vectors to deliver the all-in-one CRISPR/Cas9 expression cassette together with the on-/off-target reporter constructs (Figure S1A). In this assay, on-target activity was measured via loss of superfolder GFP (sfGFP) fluorescence, and off-target activity in the pseudogenes was analyzed via loss of sfBFP2 fluorescence. Eight days after transduction, p47.ex2 showed an on-target activity of up to 83% with a multiplicity of infection (MOI) of 1, while no significant cleavage was observed in the off-target reporter (Figures S1B–S1D). Subsequently, we proceeded with the transient delivery of CRISPR/Cas9 into iPSCs. Therefore, healthy iPSCs were nucleofected with a single-stranded oligodeoxynucleotide (ssODN) donor template and the all-in-one CRISPR/Cas9 construct, which contains a dTomato fluorescence reporter (Figures 1A and S1A). Thirty-six hours after nucleofection, CRISPR/Cas9-treated iPSCs were sorted for dTomato^+^ cells and analyzed via next-generation sequencing, which revealed about 25% of genetically modified cells (Figure 1B). The high percentage of unchanged alleles is caused by co-amplification of the pseudogenes during the PCR step, and thus represents an analysis artifact. As no cleavage of the pseudogenes is expected, about 66% of unchanged alleles originate from the pseudogene sequences. Exclusion of these sequences from the calculation to determine the percentage of modified cells led to the estimation that approximately 78% of genetically modified alleles were obtained in *NCF1*, which correlates well with our sgRNA on-target activity of 83% (Figure S1C). In the remaining alleles, 6.9% ± 1.1% had undergone non-homologous end-joining, 1.7% ± 0.3% had used the applied ssODN for homology-directed repair (HDR), and 17.5% ± 0.8% had used one of the pseudogenes as an endogenous donor template for HDR. Sequencing of the top five predicted off-target sites of p47.ex2 revealed no mutations at any of these sites (data not shown). To obtain clonal iPSC lines, we picked and screened single colonies for the desired ΔGT genotype using PCR amplification and a BsrGI restriction digest (Figures 1A and 1C). Three iPSC clones (p47-ΔGT #1, #2, and #3) were identified as correctly modified and further analyzed. Sanger sequencing confirmed the ΔGT genotype (Figure 1D). Analysis of the pluripotency markers SSEA-4 and TRA-1-60 via flow cytometry and of *OCT4*, *NANOG*, and *DNMT3B* by qPCR confirmed that pluripotency was maintained in all iPSC clones after gene editing (Figures S2A and S2B). To evaluate loss of p47^phox^ expression, we differentiated the iPSC clones into granulocytes using an embryoid body-based protocol, which gave rise to neutrophilic granulocytes (Figure 1E). Intracellular antibody staining against p47^phox^ demonstrated complete lack of p47^phox^ expression in differentiated p47-ΔGT cells (Figure 1F).

As a control, PBMCs from a p47^phox^-deficient CGD patient were reprogrammed using a 4-in-1 lentiviral vector expressing OCT4, KLF4, SOX2, and c-MYC (Figure S3A). The overall reprogramming efficiency was very low and only one iPSC clone was obtained (named p47-CGD). Sanger sequencing confirmed the ΔGT genotype (Figure S3B). The clone stained positive for SSEA-4 and TRA-1-60 and expressed the markers *OCT4*, *NANOG*, and *DNMT3B*, demonstrating pluripotency (Figures S3C and S3D). To definitively prove pluripotency, we used the p47-CGD clone in a teratoma formation assay, which gave rise to tissues of all three germ layers (Figure S3E). Finally, the clone was differentiated into granulocytes showing typical cell morphology (Figure S3F) and assessed for p47^phox^ expression. Similar to the Cas9-derived clones, p47-CGD-derived granulocytes lacked p47^phox^ expression (Figure 1F). In summary, we generated patient-like p47-ΔGT iPSCs via CRISPR/Cas9-mediated gene editing, which were genotypically and phenotypically comparable with the patient-derived p47-CGD iPSC line.

### Insertion of a Minigene into Intron 1 of *NCF1* Genetically Corrects p47^phox^ Deficiency

**Figure 2:**
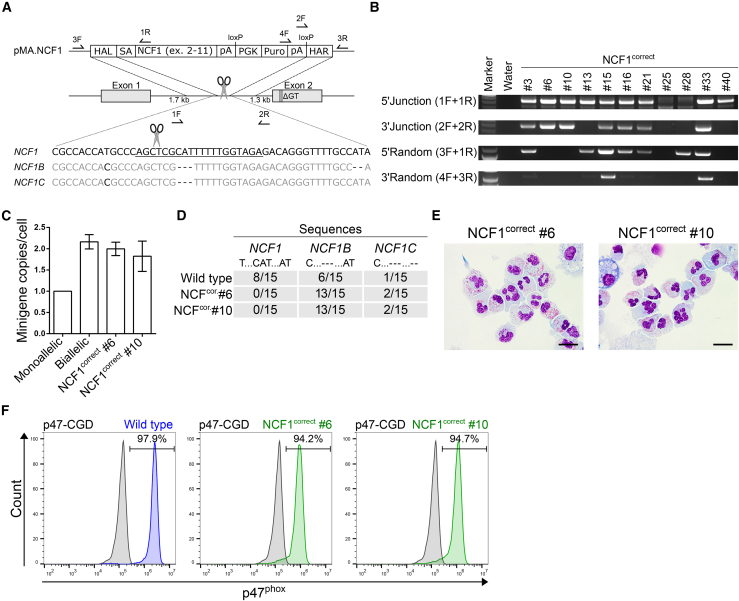
Genetic Correction of p47-ΔGT iPSCs by Targeted Insertion of a Minigene into Intron 1 of *NCF1* (A) Schematic of gene editing and correction strategy. The sgRNA target sequence (underlined) differs from the pseudogene sequence by three additional nucleotides. Arrows indicate positions of primers used for genotyping. F, forward primer; R, reverse primer; HAL, homology arm left; SA, splice acceptor site; NCF1, cDNA encoding the *NCF1* gene excluding exon 1; pA, polyadenylation signal; PGK, phosphoglycerate kinase promoter; Puro, puromycin resistance gene; HAR, homology arm right. (B) PCR-based genotyping. Applied primers were used as indicated, and their binding sites are depicted in (A). (C) Determination of the minigene copy number normalized to the *PTBP2* gene via qPCR (n = 3, mean ± SD, technical replicates). (D) Sequencing analysis of the region adjacent to the p47.in1 target site in *NCF1* and the pseudogenes. The PCR fragment was subcloned and single clones were analyzed by Sanger sequencing. Sequences were identified as *NCF1*, *NCF1B*, or *NCF1C* based on specific point mutations and deletions as indicated. (E) Cell morphology of corrected iPSC-derived granulocytes after Pappenheim staining. Scale bars, 20 μm. (F) Intracellular staining followed by flow cytometry to detect p47^phox^ expression in wild type and corrected granulocytes (gated on p47-CGD granulocytes).

The pseudogenes *NCF1B/C* are only distinguishable from *NCF1* by small-nucleotide polymorphisms, smaller insertions, and smaller deletions. Therefore, we used the sgRNA p47.in1 to target a position in intron 1 of *NCF1* that differed from the pseudogenes by three additional base pairs to avoid multiple DSB and off-target insertions of our donor construct into the pseudogenes during CRISPR/Cas9-based gene correction (Figure 2A). Our on-/off-target HT1080 reporter cell assay revealed about 80% on-target activity and lack of cleavage in the pseudogene sequence (Figures S4A–S4D). After insertion of the minigene cassette (pMA.NCF1) and selection of targeted iPSC clones via puromycin resistance, PCR screening revealed 12 out of 55 analyzed iPSC clones to be correctly targeted (28 clones had no detectable insertion at all, despite being selected arguing for insufficient puromycin selection pressure), and 2 out of these 12 clones lacked random insertion (Figure 2B). These corrected iPSC clones (NCF1^correct^ #6 and #10) had two copies of the puromycin resistance gene, which suggested a biallelic targeting (Figure 2C). Sequencing analysis of the region adjacent to the p47.in1 sgRNA target site in *NCF1* and the pseudogenes revealed a lack of the wild type *NCF1* sequence, but presence of both pseudogene sequences in both corrected clones, which provides additional confirmation of a biallelic correction in the *NCF1* locus and of a lack of cleavage activity in the pseudogenes (Figure 2D). Moreover, sequencing of the top five predicted off-target sites for p47.in1 did not reveal any mutations at these sites (data not shown). The corrected iPSC clones also stained positive for the analyzed pluripotency markers after gene correction (Figures S2C and S2D). Finally, the corrected clones were differentiated into granulocytes to analyze restoration of p47^phox^ expression (Figure 2E). As per intracellular p47^phox^ staining, both corrected clones expressed p47^phox^, although at levels slightly below wild type (Figure 2F). Application of our correction strategy to the patient-derived p47-CGD iPSC clone yielded three clones with biallelic correction out of 33 clones analyzed (Figures S3G and S3H). Differentiated granulocytes had restored p47^phox^ expression (Figures S3I and S3J). For further translation of our correction strategy, we tested the application of CRISPR/Cas9 ribonucleoprotein (RNP) complexes in combination with an adeno-associated virus (AAV) vector to deliver the donor template. In a semi-quantitative PCR, we observed successful gene correction of our iPSCs and achieved similar gene-editing rates compared with our plasmid transfection approach (Figure S4E). Taken together, insertion of a minigene cassette into intron 1 of *NCF1* corrected p47^phox^ deficiency by restoring p47^phox^ expression almost to wild type levels.

### Corrected iPSC-Derived Granulocytes Reveal Functional NADPH Oxidase Activity

**Figure 3:**
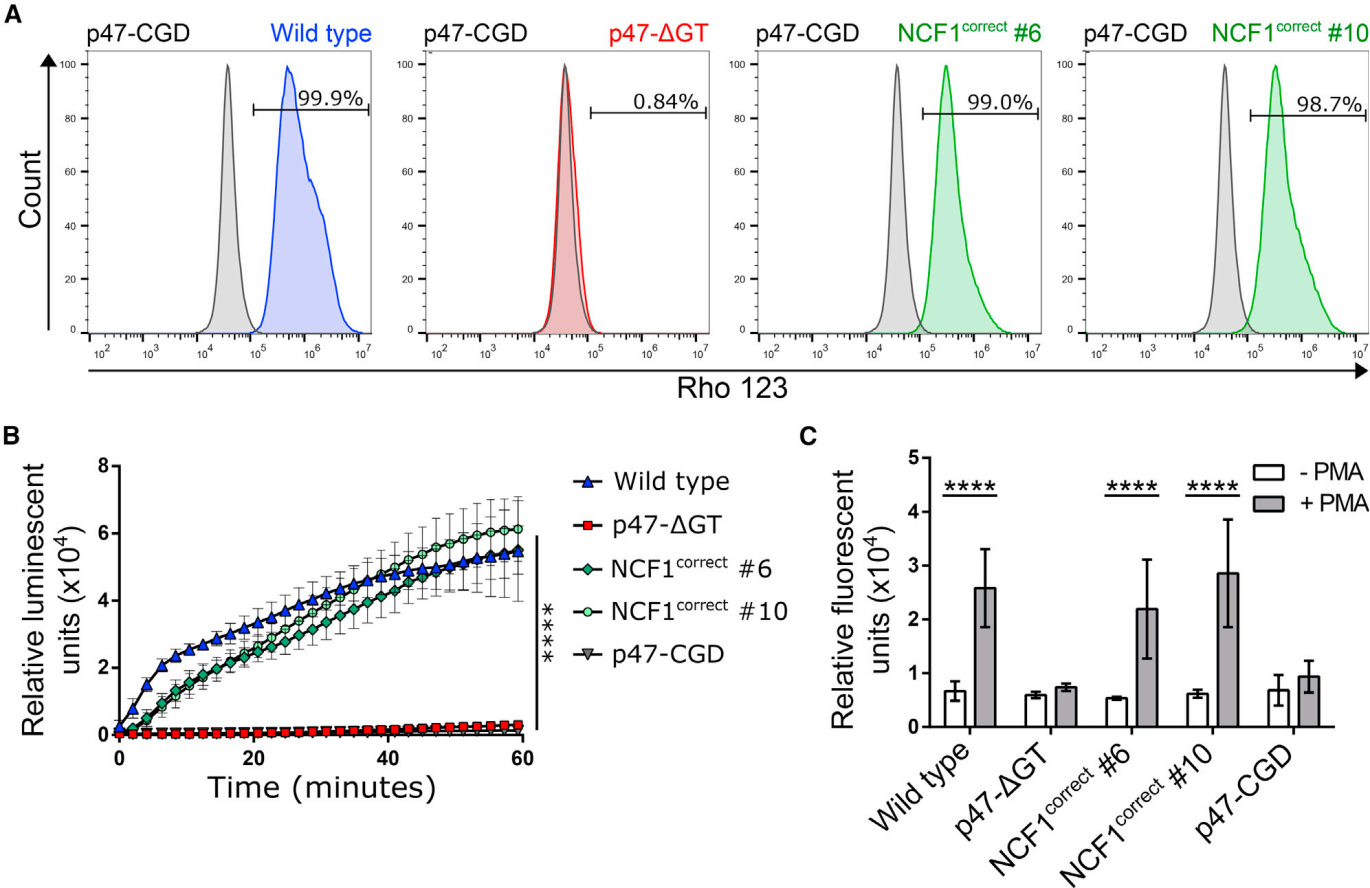
Functional Characterization of Corrected iPSC-Derived Granulocytes (A) DHR assay. After PMA stimulation, DHR is converted in the presence of ROS to green fluorescent Rho123 and assessed by flow cytometry (gated on stimulated p47-CGD granulocytes). (B) Chemiluminescent ROS assay. ROS production was measured in the presence of luminol over time in granulocytes after PMA stimulation (n = 4–7 pooled from three independent experiments; mean ± SD, one-way ANOVA; ^∗∗∗∗^p ≤ 0.0001). (C) NET formation assay. NET formation was quantified by Sytox green staining 2 h after PMA stimulation and compared with unstimulated granulocytes (n = 3–9 pooled from three independent experiments; mean ± SD, two-way ANOVA; ^∗∗∗∗^p ≤ 0.0001). Significance is only shown for stimulation.

To analyze the functional capacity of corrected granulocytes, we performed a dihydrorhodamine (DHR) assay. The readout relies on the conversion of DHR to green fluorescent Rho123 in the presence of ROS. Corrected granulocytes were 98.7%–99.0% positive for Rho123 after phorbol 12-myristate 13-acetate (PMA) stimulation, which indicated a strong oxidative burst similar to that measured for wild type granulocytes (Figures 3A and S3K). In contrast, Cas9-created p47-ΔGT granulocytes and patient-derived p47-CGD granulocytes failed to convert DHR to Rho123, confirming the lack of NADPH oxidase activity. Similarly, upon PMA stimulation, wild type and corrected granulocytes produced comparable amounts of ROS over time as measured by oxidation of luminol, while p47-ΔGT and p47-CGD granulocytes were inactive (Figure 3B). Another characteristic of neutrophilic granulocytes is the formation of NETs, which are positively regulated by ROS. NETs consist of chromatin and antimicrobial proteins that are ejected from the cell to trap and kill microbes extracellularly. Upon stimulation of differentiated granulocytes with PMA, the amount of NETs was quantified using fluorescent Sytox green (Figure 3C). The results confirmed that the level of ROS in the corrected cells was sufficient to induce NET formation at efficiencies similar to those of healthy granulocytes, while p47-ΔGT and p47-CGD granulocytes were unable to form NETs. In conclusion, we demonstrated that restored p47^phox^ expression in corrected granulocytes resulted in functional NADPH oxidase activity, ROS production, and NET formation.

### Corrected iPSC-Derived Macrophages Kill Phagocytosed Bacteria

**Figure 4:**
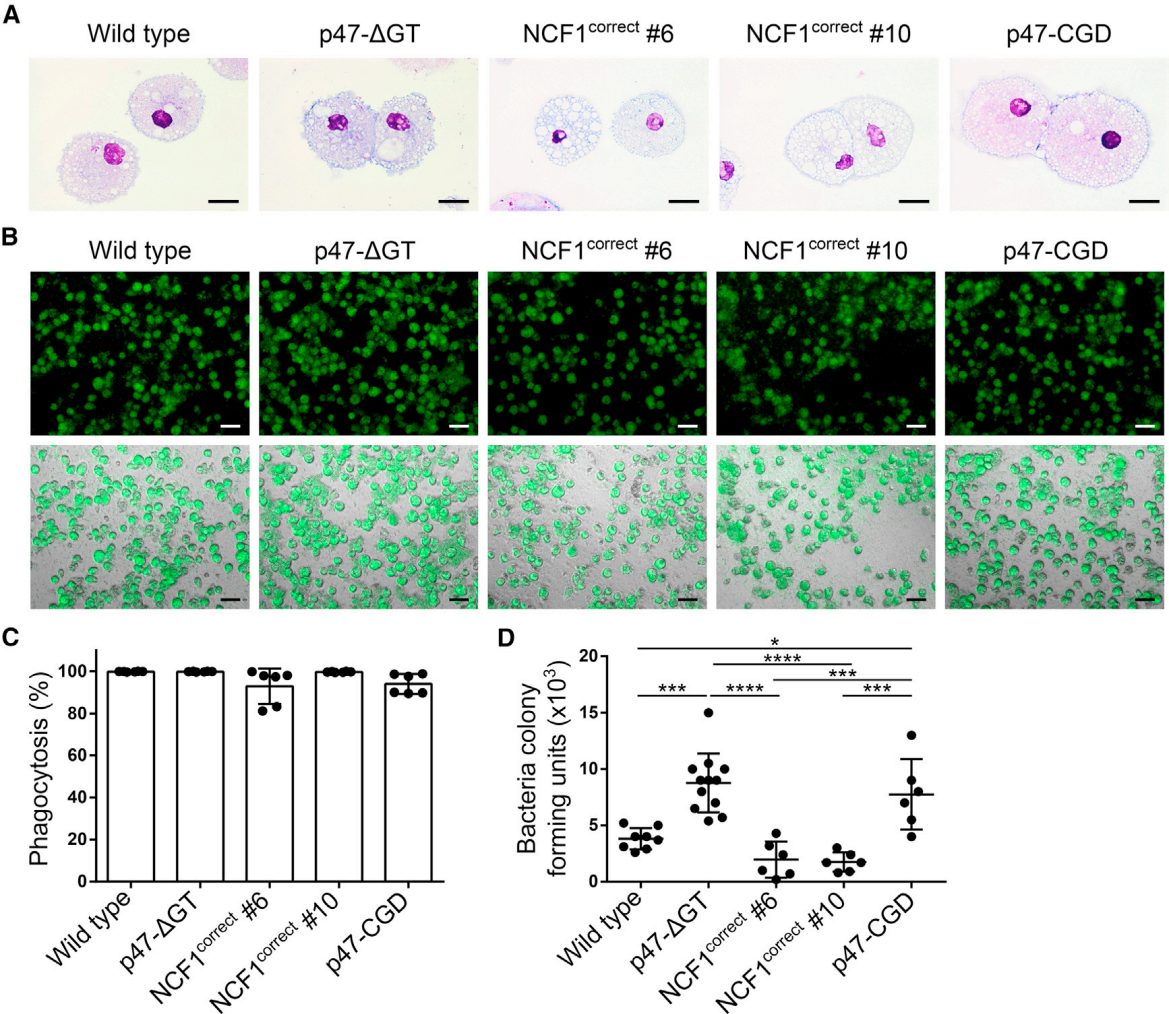
Corrected iPSC-Derived Macrophages Kill Phagocytosed Bacteria (A) Cell morphology of iPSC-derived macrophages after Pappenheim staining. Scale bars, 20 μm. (B–D) *E*. *coli* killing assay. (B) Macrophages are infected with sfGFP-labeled *E*. *coli* at an MOI of 1. Shown are microscopic pictures of macrophages that phagocytosed bacteria. Top panel: sfGFP; bottom panel: bright-field + sfGFP (scale bars, 50 μm). (C) Phagocytosis rate of total macrophages measured 6 h after infection with sfGFP-labeled *E*. *coli* by flow cytometry (n = 6 pooled from three independent experiments; mean ± SD, one-way ANOVA—no significance). (D) Colony-forming units of *E*. *coli* bacteria isolated from cell lysates of infected macrophages 24 h after infection (n = 6–12 pooled from three independent experiments; mean ± SD, one-way ANOVA; ^∗^p ≤ 0.05, ^∗∗∗^p ≤ 0.001, ^∗∗∗∗^p ≤ 0.0001).

In addition to granulocytes, the function of macrophages is also compromised in CGD patients. To investigate the benefit of the genetic correction, we differentiated our iPSC clones into macrophages. The cells showed typical macrophage morphology in Pappenheim staining (Figure 4A). To demonstrate that the differentiated macrophages were able to kill phagocytosed bacteria, we infected macrophage cultures with sfGFP-labeled *Escherichia coli* and analyzed the amount of living bacteria present inside the macrophages after 24 h of incubation. The bacterial load was determined by plating cell lysates and scoring of colony-forming units. Over 90% of all differentiated macrophages were able to phagocytose sfGFP-positive *E*. *coli* (Figures 4B and 4C). The killing assay clearly demonstrated that wild type and corrected macrophages were able to kill phagocytosed *E*. *coli* via their respiratory burst, whereas p47-ΔGT and p47-CGD macrophages retained significantly more living bacteria inside their phagosomes (Figure 4D). Furthermore, the NADPH oxidase activity appeared to be necessary for macrophages to kill phagocytosed bacteria and thus to enable the clearance of infections.

## Discussion

Targeted repair of the p47-ΔGT mutation in CGD cells is a challenging task for gene editing by CRISPR/Cas9 due to the presence of two highly homologous pseudogenes, which also carry the most frequent disease-causing mutation (ΔGT) (Roesler et al., 2000). The high sequence homology can cause unwanted off-target DSB in the pseudogenes by the Cas9. The repair of multiple breaks can then lead to larger deletions, inversions, and translocations, and thus, potentially, to chromosomal instability. In this study, we used CRISPR/Cas9 to generate a p47-ΔGT iPSC line for subsequent gene therapy development. Upon introduction of the ΔGT mutation into exon 2 of healthy iPSCs, we observed that CRISPR/Cas9 exhibited a 10-fold higher preference for using the pseudogenes as endogenous donors compared with the exogenously applied donor template. The resultant reintroduction of the pseudogene sequence into the target site via recombination could reduce the overall gene-correction frequency when applying gene editing in autologous HSCs to treat p47^phox^ deficiency.

Besides the fact that the pseudogenes carry the ΔGT mutation and serve as an endogenous donor template, the functional role of the pseudogenes is poorly understood. Whether the pseudogenes might act, for example, as non-coding RNAs that affect the transcriptome remains to be elucidated. Still contradictory, some studies indicate that certain pseudogene transcripts serve as a modulator to balance superoxide production (Xu et al., 2015) and that there might be a correlation of the ΔGT/GTGT ratio with diseases, such as inflammatory bowel disease or the onset of autoimmunity (Greve et al., 2008, Harbord et al., 2003). Thus, we decided to develop a CRISPR/Cas9 gene-correction approach for p47^phox^ deficiency that avoids cleavage of the pseudogene sequences. We identified a unique sgRNA target sequence in intron 1 of *NCF1* that allows specific insertion of our *NCF1* minigene and, thus, correction of all downstream *NCF1* mutations. We demonstrated that the corrected iPSC-derived granulocytes have restored p47^phox^ expression similar to wild type levels. Moreover, the insertion did not result in the disruption of any enhancer elements in intron 1 needed for expression as shown by comparable p47^phox^ expression levels. In a similar approach, Sweeney et al. (2017) inserted a minigene into exon 1 of *CYBB* (encoding gp91^phox^) to treat X-CGD. In their study, however, the insertion did not result in the expression of the therapeutic gp91^phox^. This experiment indicates that there may be non-coding elements in intron 1 of *CYBB* necessary for expression from the *CYBB* promoter.

To continue, we explored the use of iPSCs to recapitulate the pathogenesis of p47-CGD and develop a gene therapy strategy. The major advantages of iPSCs are that they represent an unlimited source of stem cells. Furthermore, they offer the possibility of detailed clonal analysis and selection prior to expansion and therapeutic application. Our functional assays demonstrated that the corrected iPSC-derived granulocytes and macrophages display all the features necessary to fight infections: sufficient ROS levels, induction of NET formation, and the ability to kill bacteria. Some CGD patients, who suffer from severe infections, benefit from granulocyte transfusions. However, this treatment is complicated by alloimmunization and may be detrimental for subsequent allogeneic HSCT (Connelly et al., 2018). Beyond their value for disease modeling, corrected iPSC-derived neutrophils and macrophages could be used as alternative autologous cell sources to combat intractable infections in CGD patients. In two different studies, iPSC-derived neutrophils or macrophages were successfully applied to treat bacterial infections of the peritoneum or the lung (Trump et al., 2019, Ackermann et al., 2018). Recently, the application of CRISPR-RNPs in combination with an AAV6 to deliver the donor template has yielded high HDR rates (60%–70%) during gene editing of HSCs (Vakulskas et al., 2018). We obtained preliminary data that we can induce HDR in our iPSC disease model via RNP/AAV delivery, and we are confident that the technique is transferable for application in patient-derived HSCs. However, it remains to be tested whether these tools could result in a sufficient amount of corrected granulocytes (∼5%–20%) to protect patients from severe infections (Marciano et al., 2017b).

Our study provides proof of concept for a successful CRISPR/Cas9-mediated gene correction of p47^phox^ deficiency. The functionality of the effector cells was restored, which provides the basis for gene and cell therapy treatments, such as iPSC-derived granulocyte transfusions. Much progress has been made in the last years to develop safer therapies for CGD patients, including gene therapy. This progress converted CGD from a formerly fatal disease of childhood into a disease with elaborated therapy options and a longer life expectancy.

## Experimental Procedures

### Gene Editing of iPSCs

Genetic modification was performed via nucleofection of CRISPR/Cas9 components using the Amaxa Nucleofector II and the mouse ES Cell Nucleofection Kit according to the manufacturer (both Lonza). For disease modeling, 2 × 10^6^ healthy iPSCs were nucleofected with 2.5 μg of CRISPR/Cas9 plasmid (sgRNA p47.ex2 + Cas9.2A.dTomato) and 100 pmol of a 120-bp ssODN as donor template. After fluorescence-activated cell sorting (FACSAria Fusion, Becton Dickinson) for dTomato^+^ iPSCs, colonies were expanded from single cells and analyzed (see Supplemental Information).

For genetic correction, 2 × 10^6^ p47^phox^-deficient iPSCs (p47-ΔGT #1) were nucleofected in the presence of 2.5 μg of CRISPR/Cas9 plasmid (sgRNA p47.in1 + Cas9.2A.dTomato) and 2.5 μg of the homology donor pMA.NCF1. Two days after nucleofection, iPSCs were selected using 0.2 μg/mL puromycin (InvivoGen) on multiresistant mouse embryonic feeder cells (strain DR4) kindly provided by T. Cantz (Hannover Medical School). Single iPSC colonies were analyzed for correct targeting.

Further experimental procedures are provided in Supplemental Experimental Procedures.

## Author Contributions

D.K.: conception and design, data collection, analysis and interpretation, manuscript writing. E.C., F.P., and J.D.: performed experiments. A. Selich: next-generation sequencing data analysis. R.E.S.: provided patient material. H.B.: provided vector material, conceptual advice, discussed results. J.W.S., D.H.: provided conceptual advice, discussed results. A.J.T.: discussed results, data analysis, data interpretation. A. Schambach: conception and design, financial support, data analysis, data interpretation. All authors contributed to manuscript preparation and approved the final version of the manuscript.

## Supplemental Information

Document S1. Supplemental Experimental Procedures, Figures S1–S4, and Table S1

Document S2. Article plus Supplemental Information
