# Supplementary material for: Targeted Repair of p47-CGD in iPSCs by CRISPR/Cas9: Functional Correction without Cleavage in the Highly Homologous Pseudogenes
Source: Stem Cell Reports. 2019 Sep 19;13(4):590–8. doi: 10.1016/j.stemcr.2019.08.008 (PMC6829751; doi:10.1016/j.stemcr.2019.08.008)
Supplement: Document S1. Supplemental Experimental Procedures, Figures S1–S4, and Table S1 [file mmc1.pdf]

Stem Cell Reports, Volume *13*

Supplemental Information

Targeted Repair of p47-CGD in iPSCs by CRISPR/Cas9: Functional Correction without Cleavage in the Highly Homologous Pseudogenes

Denise Klatt, Erica Cheng, Friederike Philipp, Anton Selich, Julia Dahlke, Reinhold E. Schmidt, Juliane W. Schott, Hildegard Büning, Dirk Hoffmann, Adrian J. Thrasher, and Axel Schambach

Inventory of Supplemental Information

- Figure S1. Evaluation of p47.ex2 sgRNA on-target activity. Related to Figure 1.
- Figure S2. Gene editing of iPSCs does not alter pluripotency marker expression. Related to Figures 1 and 2.
- Figure S3. Reprogramming of patient-derived PBMCs for p47-CGD disease modeling and correction. Related to Figure 1.
- Figure S4. Evaluation of p47.in1 sgRNA on-target activity. Related to Figure 2.
- Supplemental Experimental Procedures
- Table S1. Primers/oligodeoxynucleotides used in this study. Related to Figure 1, Figure 2, Figure S2, Figure S3 and Figure S4.
- Supplemental References

Figure S1. Evaluation of p47.ex2 sgRNA on-target activity. Related to Figure 1.

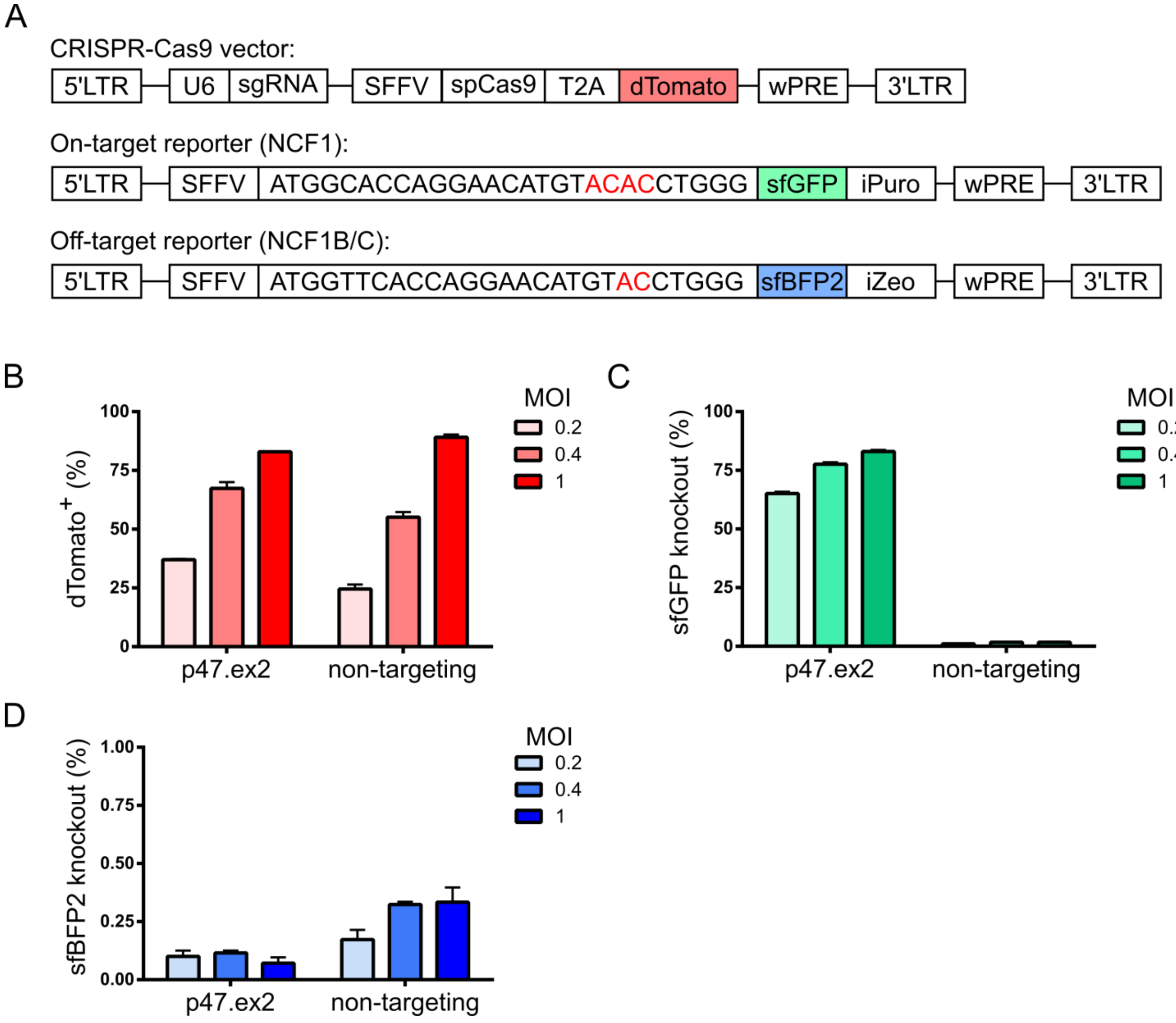

Figure S1. Evaluation of p47.ex2 sgRNA on-target activity. Related to Figure 1. **(A)** Lentiviral vectors used to evaluate sgRNA on-target activity in a HT1080 reporter cell line. The ΔGT mutation is indicated in red (antisense strand is shown). 5'LTR, 5' long terminal repeat; U6, human U6 promoter; sgRNA, single-guide RNA; SFFV, spleen focus-forming virus promoter; spCas9, *Streptococcus pyogenes*-derived Cas9; T2A, peptide cleavage site; dTomato, a red fluorescent protein; wPRE, woodchuck posttranscriptional regulatory element; 3'LTR, 3' long terminal repeat; sfGFP, super-folder green fluorescent protein; iPuro, internal ribosomal entry site (IRES, abbreviated: i) puromycin resistance gene; sfBFP2, super-folder blue fluorescent protein 2; Zeo, zeocin. **(B)** HT1080 dual on-/off-target reporter cells were transduced with the CRISPR/Cas9 vector at different MOI. Shown is the transduction rate measured as $dTomato^{+}$ cells in the total HT1080 cell population by flow cytometry (n = 3; mean ± SD). MOI, multiplicity of infection; p47.ex2, sgRNA targeting exon 2 of *NCF1*; non-targeting, non-targeting sgRNA as control. **(C)** On-target cleavage activity. Loss of sfGFP fluorescence is a surrogate marker for Cas9-mediated cleavage of the on-target reporter and was measured as $sfGFP^{-}$ cells in the $dTomato^{+}$ population by flow cytometry (n = 3; mean ± SD). **(D)** Off-target cleavage activity. Loss of sfBFP2 fluorescence is a surrogate marker for Cas9-mediated cleavage of the off-target reporter and was measured as $sfBFP2^{-}$ cells in the $dTomato^{+}$ population (n = 3; mean ± SD).

Figure S2. Gene editing of iPSCs does not alter pluripotency marker expression. Related to Figure 1 and Figure 2.

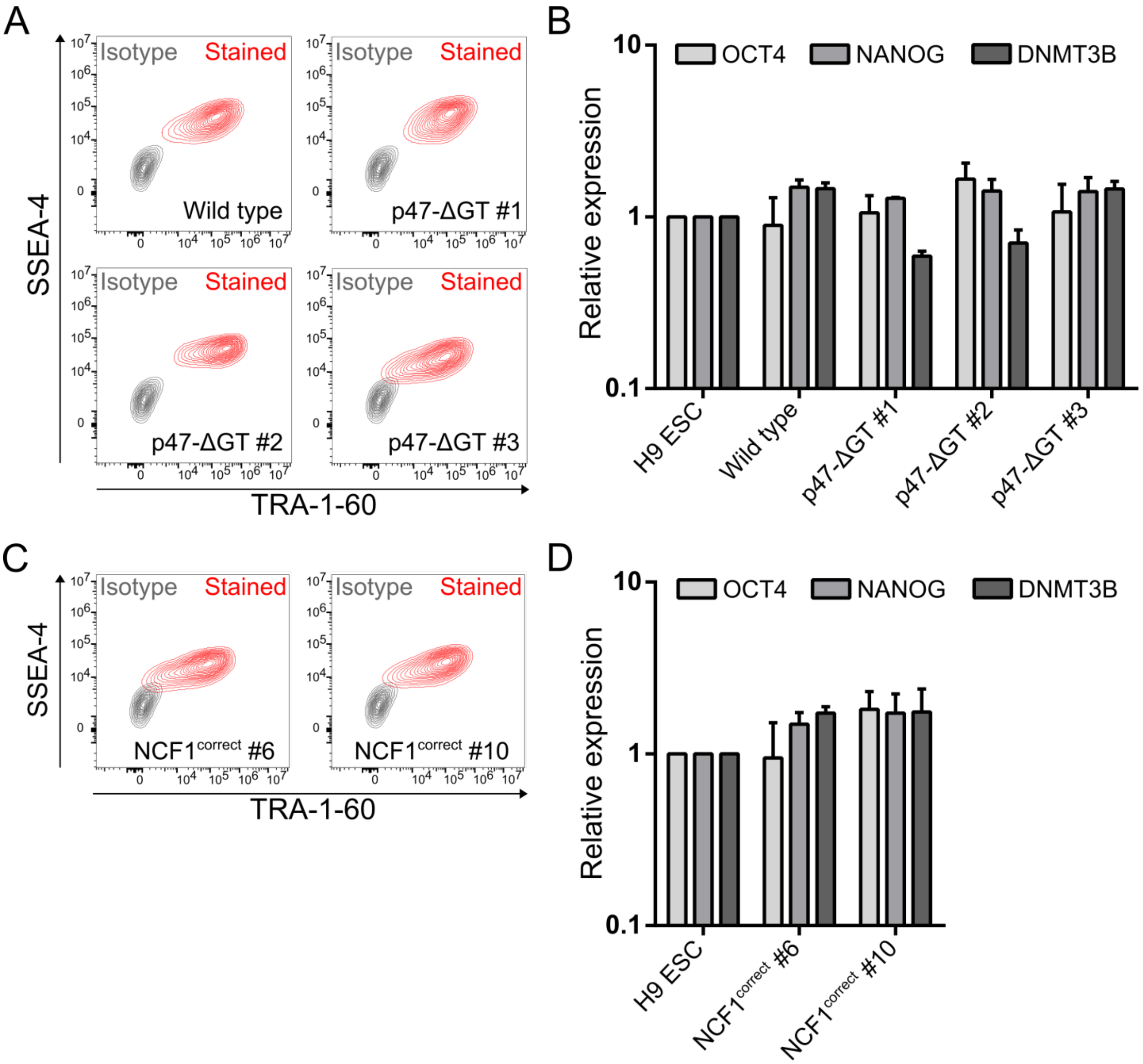

Figure S2. Gene editing of iPSCs does not alter pluripotency marker expression. Related to Figures 1 and 2. **(A, C)** Expression of the pluripotency markers SSEA-4 and TRA-1-60 measured in viable iPSCs by flow cytometry. **(B, D)** Quantitative PCR analysis assessing mRNA expression levels of *OCT4*, *NANOG* and *DNMT3B* in iPSCs normalized to *β-ACTIN* and relative to H9 embryonic stem cells (ESC).

Figure S3. Reprogramming of patient-derived PBMCs for p47-CGD disease modeling and correction. Related to Figure 1.

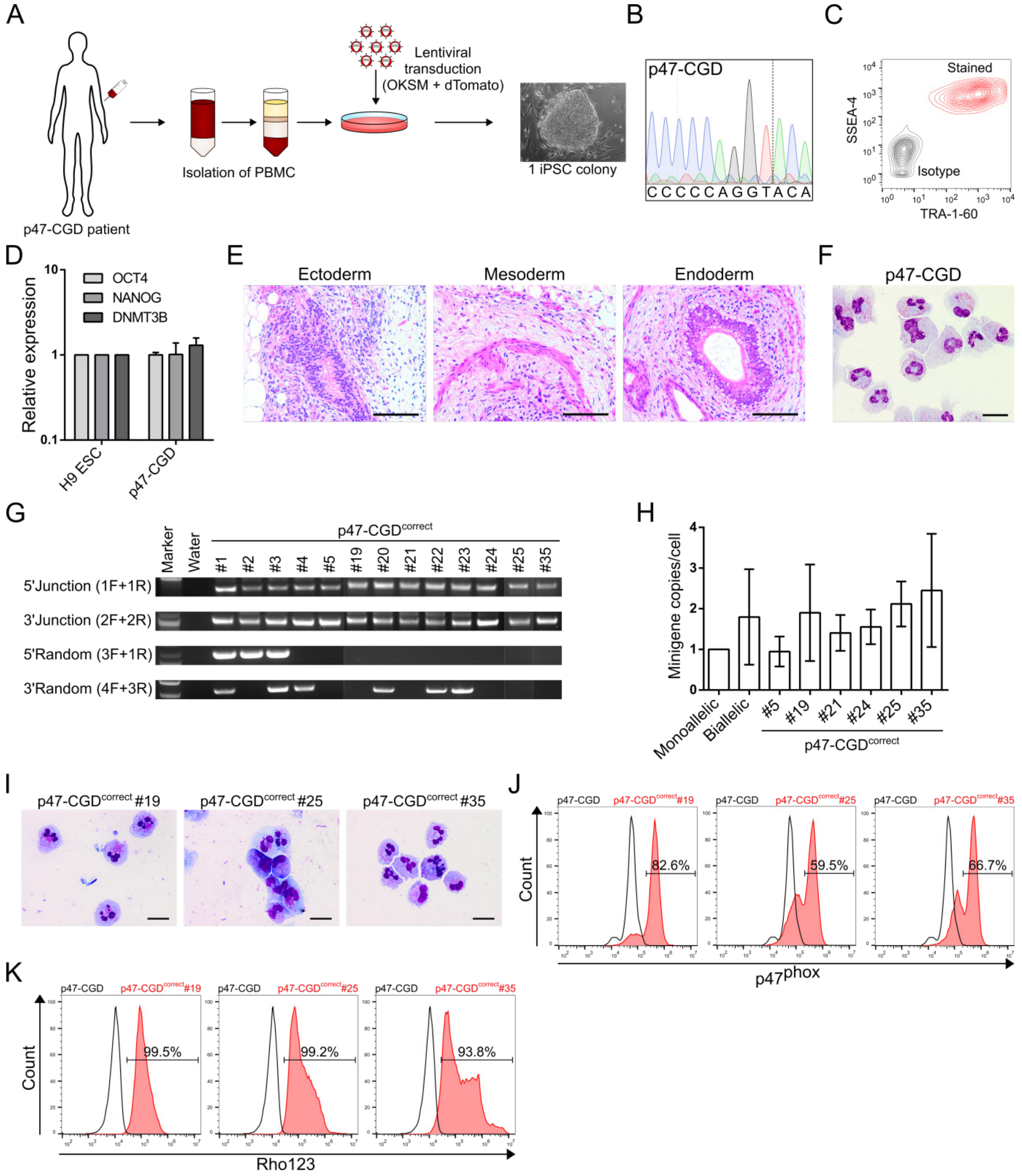

Figure S3. Reprogramming of patient-derived PBMCs for p47-CGD disease modeling and correction. Related to Figure 1. **(A)** Scheme showing reprogramming of p47-CGD patient-derived PBMCs. Isolated PBMCs were transduced with a lentiviral vector expressing the transcription factors OCT4, KLF4, SOX2 and c-MYC. Morphology of the obtained p47-CGD iPSC clone is shown. **(B)** Sanger sequencing analysis of the p47-CGD iPSC clone confirmed the presence of the ΔGT mutation. **(C)** Flow cytometric analysis of the pluripotency markers SSEA-4 and TRA-1-60 in viable iPSCs. **(D)** Quantitative PCR analysis assessing mRNA expression levels of *OCT4*, *NANOG* and *DNMT3B* in iPSCs normalized to β-*ACTIN* and relative to H9 ESCs. **(E)** Teratoma formation assay. Shown are sections from a p47-CGD iPSC-derived teratoma stained with hematoxylin eosin that represent tissues of all three germ layers (scale bar = 100 µm). **(F)** Cell morphology of p47-CGD iPSC-derived granulocytes after Pappenheim staining (scale bar = 20 µm). **(G)** PCR-based genotyping. Applied primers were used as depicted in Figure 2A. **(H)** Determination of the minigene copy number normalized to the *PTBP2* gene via qPCR (n=3, mean ± SD, technical replicates). **(I)** Cell morphology of corrected p47-CGD iPSC-derived granulocytes after Pappenheim staining (scale bar = 20 µm). **(J)** Intracellular staining followed by flow cytometry to detect $p47^{phox}$ expression in corrected p47-CGD iPSC-derived granulocytes (gated on p47-CGD granulocytes). **(K)** DHR assay of corrected p47-CGD iPSC-derived granulocytes (gated on p47-CGD granulocytes).

Figure S4. Evaluation of p47.in1 sgRNA on-target activity. Related to Figure 2.

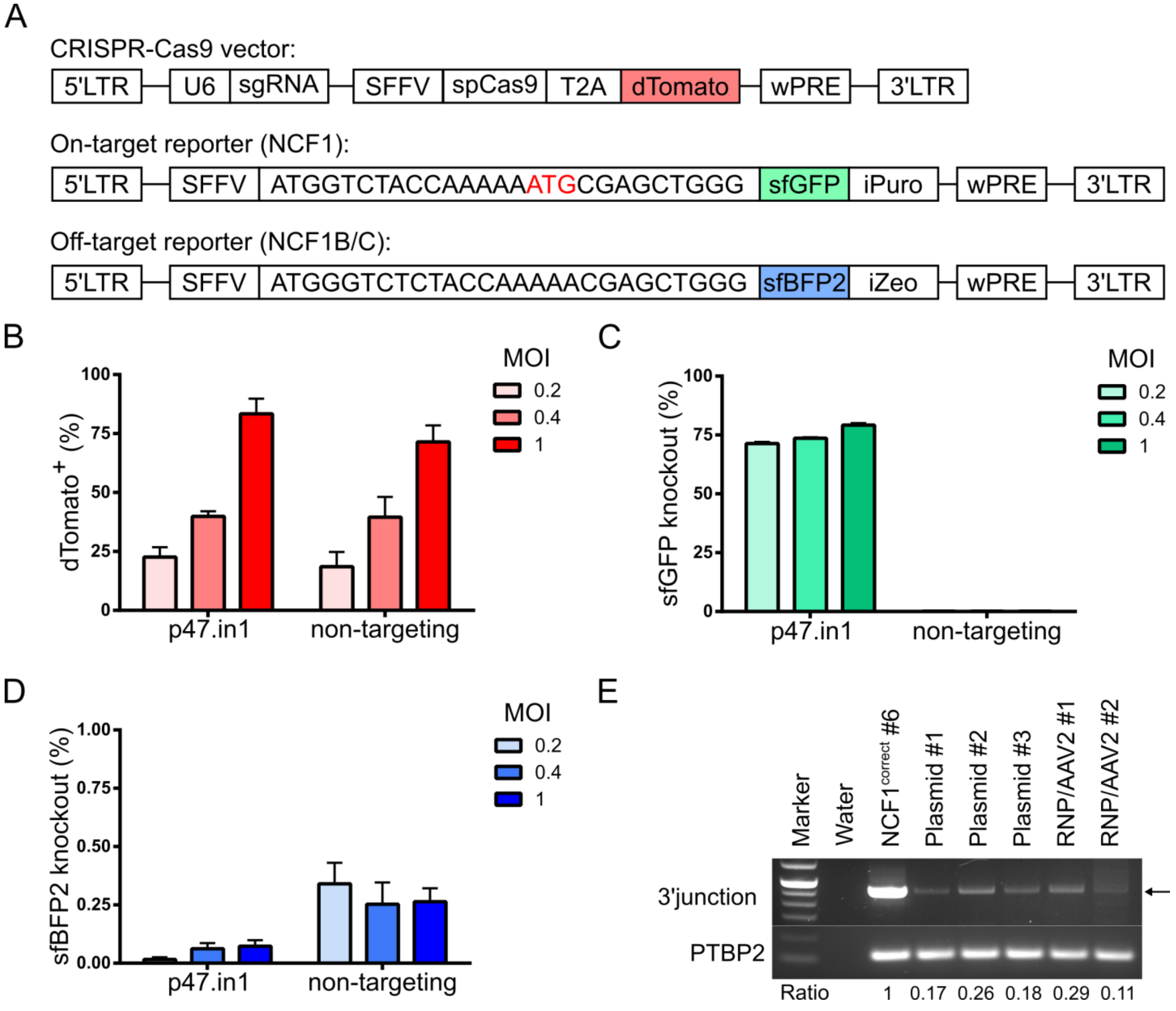

Figure S4. Evaluation of p47.in1 sgRNA on-target activity. Related to Figure 2. **(A)** Lentiviral vectors used to evaluate sgRNA on-target activity in an HT1080 reporter cell line. The three additional nucleotides are shown in red. 5'LTR, 5' long terminal repeat; U6, human U6 promoter; sgRNA, single-guide RNA; SFFV, spleen focus-forming virus promoter; spCas9, *Streptococcus pyogenes*-derived Cas9; T2A, peptide cleavage site; dTomato, a red fluorescent protein; wPRE, woodchuck posttranscriptional regulatory element; 3'LTR, 3' long terminal repeat; sfGFP, super-folder green fluorescent protein; iPuro, internal ribosomal entry site (IRES, short: i) and puromycin resistance gene; sfBFP2, super-folder blue fluorescent protein 2; Zeo, zeocin. **(B)** HT1080 dual on-/off-target reporter cells were transduced with the CRISPR/Cas9 vector at different MOI. Shown is the transduction rate measured as $dTomato^+$ cells in the total HT1080 cell population by flow cytometry (n = 3; mean ± SD). MOI, multiplicity of infection; p47.in1, sgRNA targeting intron 1 of *NCF1*; non-targeting, non-targeting sgRNA as control. **(C)** On-target cleavage activity. Loss of sfGFP fluorescence is a surrogate marker for Cas9-mediated cleavage of the on-target reporter and was measured as $sfGFP^-$ cells in the $dTomato^+$ population by flow cytometry (n = 3; mean ± SD). **(D)** Off-target cleavage activity. Loss of sfBFP2 fluorescence is a surrogate marker for Cas9-mediated cleavage of the off-target reporter and was measured as $sfBFP2^-$ cells in the $dTomato^+$ population (n = 3; mean ± SD). **(E)** Semi-quantitative PCR to assess the gene editing efficiency of the sgRNA p47.in1 using plasmid transfection and RNP/AAV2 delivery. The ratio represents the relative intensity of the 3'junction divided by PTBP2 and normalized to $NCF1^{correct}$ #6, which served as a positive control.

Supplemental Experimental Procedures

Cell culture

All iPSCs were cultivated on irradiated mouse embryonic fibroblasts C3H (MEFs; kindly provided by T. Cantz, Hannover Medical School) in F12/DMEM medium (Gibco) supplemented with 20% knockout serum replacement (Gibco), 100 U/ml penicillin and 100 µg/ml streptomycin (PAA), 2 mM L-glutamine (Biochrom), 1% non-essential amino acids (Gibco), 0.1 mM β-mercaptoethanol (Sigma-Aldrich) and 20 ng/ml β-FGF (kindly provided by the Department of Technical Chemistry, Leibniz University Hannover). Colonies were picked once per week onto new MEFs in the presence of 10 µM Y-27632 (Tocris). MEFs were seeded in low-glucose DMEM (PanBiotech) supplemented with 15% FCS (Brazil One, PanBiotech), 100 U/ml penicillin and 100 µg/ml streptomycin, 2 mM L-glutamine, 1% non-essential amino acids and 0.1 mM β-mercaptoethanol onto coated plates using 0.1% bovine gelatin (Sigma Aldrich) for 30 min.

Plasmids

An all-in-one CRISPR/Cas9 plasmid was designed to perform gene editing. The Cas9 endonuclease derived from *Streptococcus pyogenes* was cloned behind a spleen focus-forming virus (SFFV) promoter followed by a T2A.dTomato cassette to identify transfected/transduced cells. The human U6 promoter was inserted to drive expression of the respective sgRNA. Two *BsmBI* recognition sites, which are separated by a spacer sequence, were cloned in front of the sgRNA scaffold. The orientation of the two BsmBI recognition sites allows an easy introduction of phosphorylated and annealed oligodeoxynucleotides to insert the 20 nt target sequence directly in frame in front of the sgRNA scaffold. Phosphorylation was accomplished for 45 min at 37°C with T4 polynucleotide kinase (NEB) using 100 pmol each of oligodeoxynucleotides 5'p47.ex2 and 3'p47.ex2 for sgRNA p47.ex2 and of oligodeoxynucleotides 5'p47.in1 and 3'p47.in1 for sgRNA p47.in1, respectively (Table S1). Annealing was performed by incubation at 98°C for 150 s followed by a cool down to 22°C at a rate of -0.1°C/min.

For gene correction, a donor construct (pMA.NCF1) was designed carrying the *NCF1* cDNA spanning exons 2 to 11, including the endogenous splice acceptor site in front of exon 2. Behind the polyadenylation signal of the *NCF1* cassette, a loxP-site flanked puromycin selection cassette was added driven by the phosphoglycerate kinase (PGK) promoter. The whole construct is flanked by two 700 bp homology arms, which were generated via PCR amplification from the genome of the parental iPSC clone using primers HAL_fw and HAL_rev or HAR_fw and HAR_rev, respectively (Table S1).

On-/off-target reporter cell assay

To evaluate the performance of our sgRNAs (p47.ex2 and p47.in1), we designed a dual-fluorescent reporter. Therefore, the 20 nt sgRNA target sequence plus the PAM motif for each on-target site (p47.ex2: ATGGCACCAGGAACATGTACACCTGGG, p47.in1: ATGGTCTACCAAAAAATGCGAGCTGGG) were amplified via PCR in frame with and in front of a superfolder green fluorescent protein (sfGFP) and cloned into a lentiviral backbone behind an SFFV promoter. An IRES.Puromycin selection cassette was inserted behind the sfGFP. For the off-target reporter, the respective sequence of the pseudogenes (p47.ex2: ATGGTTCACCAGGAACATGTACCTGGG, p47.in1: ATGGGTCTCTACCAAAAACGAGCTGGG) was amplified via PCR in frame with and in front of a superfolder enhanced blue fluorescent protein (sfBFP2) and cloned into the lentiviral backbone, followed by an IRES.Zeocin cassette for selection. Virus production was performed using a split packaging system as described before (Maetzig *et al.*, 2014). HT1080 cells were co-transduced in the presence of 4 µg/ml protamine sulfate (Sigma Aldrich) with both reporter constructs and selected via 5 µg/ml Puromycin and 100 µg/ml Zeocin. Upon transduction of the reporter cell line with the CRISPR/Cas9 vectors, sgRNA on-/off-target efficiency was assessed as loss of fluorescence using flow cytometry (LSRII, BD).

CRISPR/Cas9 ribonucleoprotein and AAV2 donor delivery

The AAV2 donor was generated by cloning the *NCF1* minigene cassette between the inverted terminal repeats of the plasmid pSUB201 (Samulski *et al.*, 1989) via blunt end ligation. Vector production and purification was performed as described elsewhere using the newly cloned AAV plasmid instead of pAAV/EGFP (Hacker *et al.*, 2005). Genomic titer of vector preparation was determined by qPCR using donor DNA specific primers (Puro_fw and Puro_rev). For RNP formation, 300 pmol sgRNA (Synthego) were incubated with 40 pmol Alt-R® S.p. Cas9 Nuclease V3 (Integrated DNA Technologies) for 10 min at room temperature in a total volume of 5 µl and nucleofected in $5x10^5$ p47-ΔGT iPSCs as described before. The cells were seeded on a Geltrex-coated 12-well plate, incubated for 20 min at 37°C and transduced with the AAV2 vector at a particle-per-cell ratio of 10,000.

Genetic analyses

DNA was isolated from iPSCs using the QIAamp DNA Blood Mini Kit (Qiagen). To analyze the overall gene editing frequency in nucleofected and sorted iPSCs, exon 2 of *NCF1* was amplified using the MyFi PCR mix (BioLine) with primers NGS_fw and NGS_rev. In a second PCR, each sample was amplified using distinct index primers as described elsewhere (Selich *et al.*, 2016). All PCR products were pooled and sequenced using ion torrent next generation sequencing. Deep sequencing results were screened for index primers to assign sequences to the different samples with a custom Perl 5 script (https://www.perl.org). In a second step, sequences were trimmed with the flanking parts "GGCTGAATG" and "CAGGACCTGT" embedding a region of interest. The alignment was performed with a custom R (https://www.R-project.org/) script, employing the packages Biostrings and msa (Pages *et al.*, 2018; Bodenhofer *et al.*, 2015).

To identify single p47-ΔGT iPSC clones, the Phire Hot Start II DNA Polymerase (Thermo Scientific) was used to amplify exon 2 using primers p47.2_fw and p47.2_rev (543 bp amplicon). PCR products were isolated from a 1% agarose gel (Invitrogen) and digested for 30 min with the restriction enzyme BsrGI (NEB). The wild type *NCF1* sequence was cleaved by BsrGI, which resulted in 362 bp and 181 bp fragments. The GT-deletion caused a loss of the BsrGI recognition site; thus, the lack of cleavage products indicated loss of the wild type sequence. Positively identified clones were confirmed by Sanger sequencing (Seqlab).

To verify genetically corrected clones, all PCRs were performed using the Phusion Green Hot Start II High-Fidelity PCR Master Mix (Thermo Scientific). To detect the 5'junction of the inserted donor construct primers 1F and 1R were used (850 bp amplicon). The 3'junction was amplified using primers 2F and 2R (801 bp amplicon). Random insertion of the plasmid was addressed by amplifying the 5' and 3' ends of the plasmid with primers 3F/1R (1148 bp amplicon) and 4F/3R (1232 bp amplicon), respectively. All PCR products were analyzed by 1% agarose gel electrophoresis.

Intact pseudogene sequences were analyzed by amplification of wild type sequences around the Cas9 cleavage site in a nested PCR using primers W1F and W1R (481 bp amplicon) and W2F and W2R (194 bp amplicon) and the Phusion Green Hot Start II High-Fidelity PCR Master Mix. After Sanger sequencing of the extracted PCR fragments, sequences were identified based on the presence or absence of specific indels and assigned to *NCF1*, *NCF1B* or *NCF1C*. All primers are listed in Table S1.

Minigene copy number determination by quantitative PCR (qPCR)

Genomic DNA was isolated from corrected clones using the QIAamp DNA Blood Mini Kit. Primers specific for the puromycin resistance gene (Puro_fw and Puro_rev) were used to determine the minigene copy number and are described in Table S1. The qPCR was performed in triplicates with the Step One Plus Real Time PCR System (Applied Biosystems, Life Technologies) and the QuantiTect SYBR Green PCR Kit (Qiagen) according to the manufacturer's manual. The copy number of the puromycin resistance gene was normalized to *PTBP2* levels.

Generation of patient-derived $p47^{phox}$-deficient (p47-CGD) iPSCs

The human blood sample was collected after written informed consent of the donor at Hannover Medical School (Germany). Peripheral blood mononuclear cells (PBMCs) were isolated from 9 ml peripheral blood using a ficoll gradient (Biochrom). Cells were reprogrammed into iPSCs as described elsewhere (Warlich *et al.*, 2011). Briefly, 2×10^5 cells were transduced with a lentiviral vector expressing the four transcription factors OCT4, KLF4, SOX2 and c-MYC in the presence of 8 µg/ml polybrene (Sigma Aldrich). The cells were cultured in HSC medium consisting of StemSpan (STEMCELL Technologies) supplemented with 100 U/ml penicillin and 100 µg/ml streptomycin, 100 ng/ml stem cell factor, 50 ng/ml thrombopoietin and 100 ng/ml fms-like tyrosine kinase 3 (all cytokines: Peprotech). The day after transduction, 50 µg/ml 2-phospho-L-ascorbic acid (Sigma-Aldrich) and 2 mM valproic acid (Ergenyl®, Sanofi-Aventis) were added to the medium. Three days after transduction, cells were transferred onto MEFs and cultured in HSC and F12/DMEM-iPSC medium in a mixture of 1:1. Medium was changed twice a week using F12/DMEM-iPSC medium. After three to four weeks, one iPSC colony formed and was analyzed for pluripotency.

Pluripotency assays

All iPSCs were analyzed for TRA-1-60 and SSEA-4 expression using flow cytometry. Therefore, iPSCs were trypsinized, resuspended into single cells, blocked with human FcR block (Miltenyi) for 10 min and stained with TRA-1-60-PE (Miltenyi) and SSEA-4-Alexa Fluor 647 (BD) for 30 min at 4°C. Data was acquired using a CytoFlex S flow cytometer (Beckman Coulter).

Expression levels of the pluripotency markers *NANOG*, *OCT4* and *DNMT3B* were analyzed via qPCR. Therefore, RNA was isolated from each iPSC clone using the RNeasy Mini Kit (Qiagen) and reverse transcribed into cDNA using the QuantiTect Reverse Transcription Kit (Qiagen) as per manufacturer's instruction. The qPCR was

performed in triplicates with the Step One Plus Real Time PCR System and the QuantiTect SYBR Green PCR Kit according to the manufacturer's manual. Primer sequences for *NANOG*, *OCT4*, *DNMT3B* and *β-ACTIN* are described in Table S1. The expression level of each target gene was normalized to *β-ACTIN* expression levels and expressed in relation to the embryonic stem cell (ESC) clone H9 using the ΔΔCt method (Pfaffl, 2001).

The potential of generated iPSCs to differentiate into cells of all three germ layers was assessed in a teratoma formation assay as described before (Philipp *et al.,* 2018). Briefly, 3×10^6 iPSCs were resuspended in 100 µl F12/DMEM-iPSC medium containing 20 µM Y-27632. After addition of 100 µl Matrigel basement membrane matrix (Corning), the cell suspension was subcutaneously injected into the flanks of an NBSGW mouse (NOD.Cg-Kit^{W-41J} Tyr^{+} $Prkdc^{scid}$ $Il2rg^{tm1Wjl}$/ThomJ). The animal experiment was performed in accordance with Lower Saxony State Office for Consumer Protection and Food Safety in Germany. The mouse was sacrificed after the teratoma reached a diameter of 1.5 cm. After isolation and fixation of the teratoma in 4% buffered formaldehyde (Carl Roth), the tissue was embedded in paraffin, sectioned into 3 µm slices and stained with hematoxylin and eosin. Microscopic analysis was performed with an Olympus BX51 microscope (software Cell^F version 3.4, Olympus).

Myeloid differentiation of iPSCs

Differentiation of iPSCs into granulocytes and macrophages was performed using an embryoid body (EB)-based protocol as previously described (Lachmann *et al.*, 2015). Briefly, iPSC colonies were detached using dispase (Roche) and transferred into EB medium (KO/DMEM medium (Gibco) supplemented with 20% knockout serum replacement (Gibco), 100 U/ml penicillin and 100 µg/ml streptomycin (PAA), 2 mM L-glutamine (Biochrom), 1% non-essential amino acids (Gibco), 0.1 mM β-mercaptoethanol (Sigma-Aldrich) and 10 µM Y-27632 (Tocris)). EB formation was done for 5 days on an orbital shaker at 80 rpm. For granulocyte differentiation, EBs were picked into APEL2 medium (StemCell Technologies) supplemented with 100 U/ml penicillin and 100 µg/ml streptomycin, 50 ng/ml human granulocyte colony-stimulating factor (G-CSF, Peprotech) and 25 ng/ml human interleukin-3 (Peprotech). For macrophage differentiation, EBs were transferred into X-Vivo15 medium (Lonza) supplemented with 100 U/ml penicillin and 100 µg/ml streptomycin, 2 mM L-glutamine, 50 ng/ml human macrophage colony-stimulating factor (M-CSF, Peprotech) and 25 ng/ml human interleukin-3. Differentiated cells were harvested once per week and cultured in RPMI 1640 medium (PanBiotech) supplemented with 10% fetal calf serum (FCS, Brazil One, PanBiotech), 100 U/ml penicillin and 100 µg/ml streptomycin, 2 mM L-glutamine, 100 ng/ml G-CSF or M-CSF, respectively.

Intracellular $p47^{phox}$ staining

The FoxP3 intracellular staining kit (Biolegend) was used for fixation and permeabilization of differentiated granulocytes. Briefly, cells were fixed for 20 min in Fix/Perm solution, washed once with PBS and once with Perm buffer. After incubation in Perm buffer for 15 min, cells were stained with the primary $p47^{phox}$ antibody (BD) for 30 min. Secondary antibody staining was performed for 30 min using a goat-anti-mouse DyLight488 antibody (Thermo Fisher). Stained cells were analyzed using a CytoFlex S flow cytometer.

Pappenheim staining

To analyze cell morphology, 5×10^4 cells were spun onto microscope glass slides using a Cytospin 4 (Thermo Scientific), stained with May-Grünwald and Giemsa according to manufacturer's instructions (Pappenheim staining, Sigma-Aldrich) and analyzed with an Olympus BX51 microscope (software Cell^F version 3.4, Olympus).

Functional granulocyte assays

The dihydrorhodamine (DHR) assay was performed as described elsewhere (Dreyer *et al.*, 2015), with minor modifications. Briefly, 10^5 cells were resuspended in 500 µl Hank's Buffered Salt Solution (HBSS) containing Mg/Ca (Gibco). After stimulation with 40 nM phorbol 12-myristate 13-acetate (PMA; Sigma Aldrich) in the presence of 1000 U catalase (Sigma Aldrich) for 5 min, 250 ng DHR (Sigma Aldrich) were added for 15 min to the cells. Cells were placed on ice to stop the reaction and analyzed by flow cytometry (CytoFlex S).

ROS production was assessed using a luminol chemiluminescence assay. Therefore, 10^5 cells were resuspended in 200 µl HBSS containing Mg/Ca, supplemented with 0.5% human albumin (Grifols) and 0.5 mM luminol (Sigma Aldrich). After stimulation with 40 nM PMA, ROS production was measured every 2 min for 60 min at 37°C at a multi-mode plate reader (Beckman Coulter).

NET formation was quantified as described before (Dreyer *et al.*, 2015), with minor modifications. For each sample, 3.5×10^4 cells were stimulated with 40 nM PMA and stained with 1 mM Sytox Green (Invitrogen) to visualize the DNA. NET formation was quantified using a fluorescence microplate reader (Berthold technologies) with a filter setting of 485/535 nm (excitation/emission).

Bacteria killing assay

Competent XL-1 blue *E. coli* bacteria were transformed with a plasmid carrying sfGFP, plated on LB-agar plates (CarlRoth) supplemented with 75 µg/ml ampicillin (Ratiopharm) and grown overnight. The next day, 10^5 macrophages were seeded in a 48-well plate in antibiotic-free medium (RPMI 1640 (PanBiotech) supplemented with 10% FCS, 2 mM L-glutamine and 100 ng/ml M-CSF). An sfGFP-positive colony was picked, transferred into 100 ml LB-medium (CarlRoth) plus ampicillin and grown to an OD_{600} of 0.5 overnight (~12-14 h). Macrophages were infected with the bacteria at an MOI of 1, and 40 µg/ml meropenem (Hexal) was added to the medium one hour post-infection. After another 24 h, all cells were harvested and lysed in ultrapure water (Biochrom) for 2 min. Serial dilutions were made in PBS (Lonza), plated on LB-agar plates supplemented with ampicillin and incubated overnight to determine the number of colony forming units. Prior to cell lysis, fluorescence microscope pictures were taken using a Zeiss Observer Z1 microscope (AxioVision software). Additional wells with macrophages were infected to measure the phagocytosis rate by flow cytometry (FACS Calibur).

Statistical tests

Statistical significance was determined using one-way or two-way ANOVA with GraphPad Prism 7. All bar graphs represent the mean ± SD. *P≤0.05, **P≤0.01, ***P≤0.001, ****P≤0.0001.

Table S1: Primers/oligodeoxynucleotides used in this study. Related to Figure 1, Figure 2, Figure S2, Figure S3 and Figure S4.

Name	Sequence (5'-3')
p47.ex2 donor (ssODN)	GAACTCGTAGATCTCGGTGAAGCGCCGGTAGACCACCTTCTCCGACAGGTCCTGC CATTTCACCAGGAACATGTACCTGGAGGAAAGCCAGAGTCGGGGGACCCCATTCA GCCTCCAAAG
5'p47.ex2	CACCGCACCAGGAACATGTACACCT
3'p47.ex2	AAACAGGTGTACATGTTCCTGGTGC
5'p47.in1	CACCGTCTACCAAAAAATGCGAGCT
3'p47.in1	AAACAGCTCGCATTTTTTGGTAGAC
HAL_fw	AGCGCTATAGATCTTGTGGAGATGAGGTTTCACT
HAL_rev	AGAGTCGGGCTAGCCATGGTGGCGCGCACCTGTA
HAR_fw	TTCTGCAGACGCGTGACAGGGTTTTGCCATATTG
HAR_rev	CTAGAGTCGCGGCCGCGCCTGACCAATATGGTGAAA
NGS_fw	GGTGGGTTTTCCAGTCACACGCCCTTTCTGCAATCCAGGACAACC
NGS_rev	TTCGTTGGGAGTGAATTAGCCTGGGTCCCTGTCCCTCCTCCA
p47.2_fw	CTGGGGCGTGGCAGCACTTGGGT
p47.2_rev	GCTATGATTGCGCCCCTGCACTGCAA
1F	ACACCACCATGCCTGGCTAGTT
1R	TAAGGTTTTATGGAACTCGTAGATCTC
2F	TACGAAGTTATTCTGCAGACGCG
2R	GTTGGCTCCCGTCTGTAATCC
3F	TAACCAATAGGCCGAAATCG
4F	GCATCGCATTGTCTGAGTAGG
3R	TATAGTCCTGTCGGGTTTCG
W1F	CCGATTATCCTGCTTGTCCTCTGCAGTG
W1R	ATGGTGGCGCATGCCTGTAATCC
W2F	AGAGATTCTCCTGCCTCAGCC
W2R	ATGCAGTGAGTGGAGATTGTGCC
NANOG_fw	TCACACGGAGACTGTCTCTC
NANOG_rev	GAACACAGTTCTGGTCTTCTG
OCT4_fw	CCTCACTTCACTGCACTGTA
OCT4_rev	CAGGTTTTCTTTCCCTAGCT
DNMT3B_fw	ATAAGTCGAAGGTGCGTCGT
DNMT3B_rev	GGCAACATCTGAAGCCATTT
β-ACTIN_fw	TACCTGTATAGTGTACTTCAT
β-ACTIN_rev	GGTCATGAGAAGTGTTGCTA
Puro_fw	CACCAGGGCAAGGGTCTG
Puro_rev	CTCGTAGAAGGGGAGGTTGC
PTBP2_fw	TCTCCATTCCCTATGTTCATGC
PTBP2_rev	GTTCCCGCAGAATGGTGAGGTG

Supplemental References

Bodenhofer U, Bonatesta E, Horejs-Kainrath C, Hochreiter S (2015). “msa: an R package for multiple sequence alignment.” Bioinformatics, 31(24), 3997–3999. doi: 10.1093/bioinformatics/btv494.

Dreyer, A., Hoffmann, D., Lachmann, N., Ackermann, M., Steinemann, D., Timm, B., Siler, U., Reichenbach, J., Grez, M., Moritz, T. et al. (2015). TALEN-mediated functional correction of X-linked chronic granulomatous disease in patient-derived induced pluripotent stem cells. Biomaterials *69*, 191-200.

Hacker, U.T., Wingenfeld, L., Kofler, D.M., Schuhmann, N.K., Lutz, S., Herold, T., King, S.B.S., Gerner, F.M., Perabo, L., Rabinowitz, J. et al. (2005). Adeno-associated virus serotypes 1 to 5 mediated tumor cell directed gene transfer and improvement of transduction efficiency. J Gene Med *7*, 1429-1438.

Lachmann, N., Ackermann, M., Frenzel, E., Liebhaber, S., Brennig, S., Happle, C., Hoffmann, D., Klimenkova, O., Lüttge, D., Buchegger, T. et al. (2015). Large-scale hematopoietic differentiation of human induced pluripotent stem cells provides granulocytes or macrophages for cell replacement therapies. Stem Cell Reports 4, 282-296.

Maetzig, T., Kuehle, J., Schwarzer, A., Turan, S., Rothe, M., Chaturvedi, A., Morgan, M., Ha, T.C., Heuser, M., Hammerschmidt, W. et al. (2014). All-in-One inducible lentiviral vector systems based on drug controlled FLP recombinase. Biomaterials *35*, 4345-4356.

Pagès H, Aboyoun P, Gentleman R, DebRoy S (2018). Biostrings: Efficient manipulation of biological strings. R package version 2.50.1.

Pfaffl, M.W. (2001). A new mathematical model for relative quantification in real-time RT-PCR. Nucleic Acids Res. 29, 45e–45.

Philipp, F., Selich, A., Rothe, M., Hoffmann, D., Rittinghausen, S., Morgan, M.A., Klatt, D., Glage, S., Lienenklaus, S., Neuhaus, V. et al. (2018). Human Teratoma-Derived Hematopoiesis Is a Highly Polyclonal Process Supported by Human Umbilical Vein Endothelial Cells. Stem Cell Reports *11*, 1051-1060.

Samulski R.J., Chang L.S., Shenk T. (1989). Helper-Free Stocks of Recombinant Adeno-Associated Viruses: Normal Integration Does Not Require Viral Gene Expression. J Virol *63*, 3822-3828.

Selich, A., Daudert, J., Hass, R., Philipp, F., von Kaisenberg, C., Paul, G., Cornils, K., Fehse, B., Rittinghausen, S., Schambach, A. et al. (2016). Massive Clonal Selection and Transiently Contributing Clones During Expansion of Mesenchymal Stem Cell Cultures Revealed by Lentiviral RGB-Barcode Technology. Stem Cells Transl Med *5*, 591-601.

Warlich, E., Kuehle, J., Cantz, T., Brugman, M.H., Maetzig, T., Galla, M., Filipczyk, A.A., Halle, S., Klump, H., Schöler, H.R. et al. (2011). Lentiviral vector design and imaging approaches to visualize the early stages of cellular reprogramming. Mol. Ther. *19*, 782-789.
