# Supplementary material for: Targeted Repair of p47-CGD in iPSCs by CRISPR/Cas9: Functional Correction without Cleavage in the Highly Homologous Pseudogenes
Source: Stem Cell Reports. 2019 Sep 19;13(4):590–8. doi: 10.1016/j.stemcr.2019.08.008 (PMC6829751; doi:10.1016/j.stemcr.2019.08.008)
Supplement: Document S2. Article plus Supplemental Information [file mmc2.pdf]

Stem Cell Reports
Report
OPEN ACCESS

Targeted Repair of p47-CGD in iPSCs by CRISPR/Cas9: Functional Correction without Cleavage in the Highly Homologous Pseudogenes

Denise Klatt,[1,2] Erica Cheng,[1,2] Friederike Philipp,[1,2,3] Anton Selich,[1] Julia Dahlke,[1,2] Reinhold E. Schmidt,[4] Juliane W. Schott,[1] Hildegard Büning,[1,2] Dirk Hoffmann,[1,2] Adrian J. Thrasher,[5,6] and Axel Schambach[1,2,7,*]
[1]Institute of Experimental Hematology, Hannover Medical School, Carl-Neuberg-Strasse 1, 30625 Hannover, Germany
[2]REBIRTH Cluster of Excellence, Hannover Medical School, 30625 Hannover, Germany
[3]Fraunhofer Institute for Toxicology and Experimental Medicine, 30625 Hannover, Germany
[4]Department of Immunology and Rheumatology, Hannover Medical School, 30625 Hannover, Germany
[5]Infection, Immunity and Inflammation Program, Molecular and Cellular Immunology Section, UCL Great Ormond Street Institute of Child Health, University College London, London WC1N 1EH, UK
[6]Great Ormond Street Hospital NHS Foundation Trust, London WC1N 1EH, UK
[7]Division of Hematology/Oncology, Boston Children's Hospital, Harvard Medical School, Boston, MA 02115, USA
*Correspondence: schambach.axel@mh-hannover.de
https://doi.org/10.1016/j.stemcr.2019.08.008

SUMMARY

Mutations in the NADPH oxidase, which is crucial for the respiratory burst in phagocytes, result in chronic granulomatous disease (CGD). The only curative treatment option for CGD patients, who suffer from severe infections, is allogeneic bone marrow transplantation. Over 90% of patients with mutations in the p47^{phox} subunit of the oxidase complex carry the deletion c.75_76delGT (ΔGT). This frequent mutation most likely originates via gene conversion from one of the two pseudogenes *NCF1B* or *NCF1C*, which are highly homologous to *NCF1* (encodes p47^{phox}) but carry the ΔGT mutation. We applied CRISPR/Cas9 to generate patient-like p47-ΔGT iPSCs for disease modeling. To avoid unpredictable chromosomal rearrangements by CRISPR/Cas9-mediated cleavage in the pseudogenes, we developed a gene-correction approach to specifically target *NCF1* but leave the pseudogenes intact. Functional assays revealed restored NADPH oxidase activity and killing of bacteria in corrected phagocytes as well as the specificity of this approach.

INTRODUCTION

Loss-of-function mutations in the NADPH oxidase cause chronic granulomatous disease (CGD), a rare primary immunodeficiency characterized by defective phagocytes unable to regulate efficient killing of engulfed pathogens by granule proteases (Reeves et al., 2002; Roos, 1994). Thus, patients suffer from recurrent bacterial and fungal infections and rely on lifelong antibiotic and antifungal prophylaxis (Seger, 2008). Despite improved control of the disease, many patients still develop severe infections that are often refractory to conventional therapy (Holland, 2010). To combat these infections, patients receive cell-based therapies, such as granulocyte transfusions or allogenic hematopoietic stem cell transplantation (HSCT) (Güngör et al., 2014; Marciano et al., 2017a). The latter is the only curative treatment option. Early efforts to treat CGD by gammaretroviral gene therapy were complicated by mutagenesis (Ott et al., 2006). More recent studies using chimeric regulatory sequences in a lentiviral vector platform are showing significant promise, although ultimately a precise gene-editing approach is an attractive option.

While the disease-causing mutations are very heterogeneous for most NADPH oxidase subunits, over 90% of patients with a mutation in the p47^{phox} subunit (encoded by *NCF1*), carry the deletion c.75_76delGT (ΔGT) in the 5′ region of exon 2 (Roos et al., 2010). The high prevalence of this mutation (25% of overall CGD patients) most likely originates via gene conversion from one of the two pseudogenes *NCF1B* and *NCF1C*, which also harbor the ΔGT mutation, have a sequence homology of over 99% compared with *NCF1*, and are in close proximity to *NCF1* (Roesler et al., 2000). The functional role of the pseudogenes is as yet not well studied. Xu et al. (2015) demonstrated that overexpression of certain pseudogene transcripts resulted in downregulation of superoxide production in endothelial cells, indicating a functional role. Moreover, Merling et al. (2017) showed that correction of the ΔGT mutation in the pseudogenes using zinc-finger nucleases restored protein expression and resulted in functional NADPH oxidase activity. However, directly targeting the ΔGT mutation carries the risk of chromosomal aberrations. Every designer nuclease, even the highly site-specific CRISPR/Cas9 system, will potentially introduce two DNA double-strand breaks (DSB) into *NCF1* and into each pseudogene due to their sequence homology. Consequently, the repair of up to six DSBs can result in larger deletions, inversions, or translocations, which could potentially cause severe, unwanted alterations in the genome.

Human induced pluripotent stem cells (iPSCs) are used in many research fields as disease models to develop novel treatment strategies. However, patient-derived cells, such as peripheral blood mononucleated cells (PBMCs) or skin fibroblasts, are needed for reprogramming, and primary material can be difficult to obtain when studying rare diseases. Here, we used CRISPR/Cas9 to generate patient-like

This is an open access article under the CC BY license (http://creativecommons.org/licenses/by/4.0/).

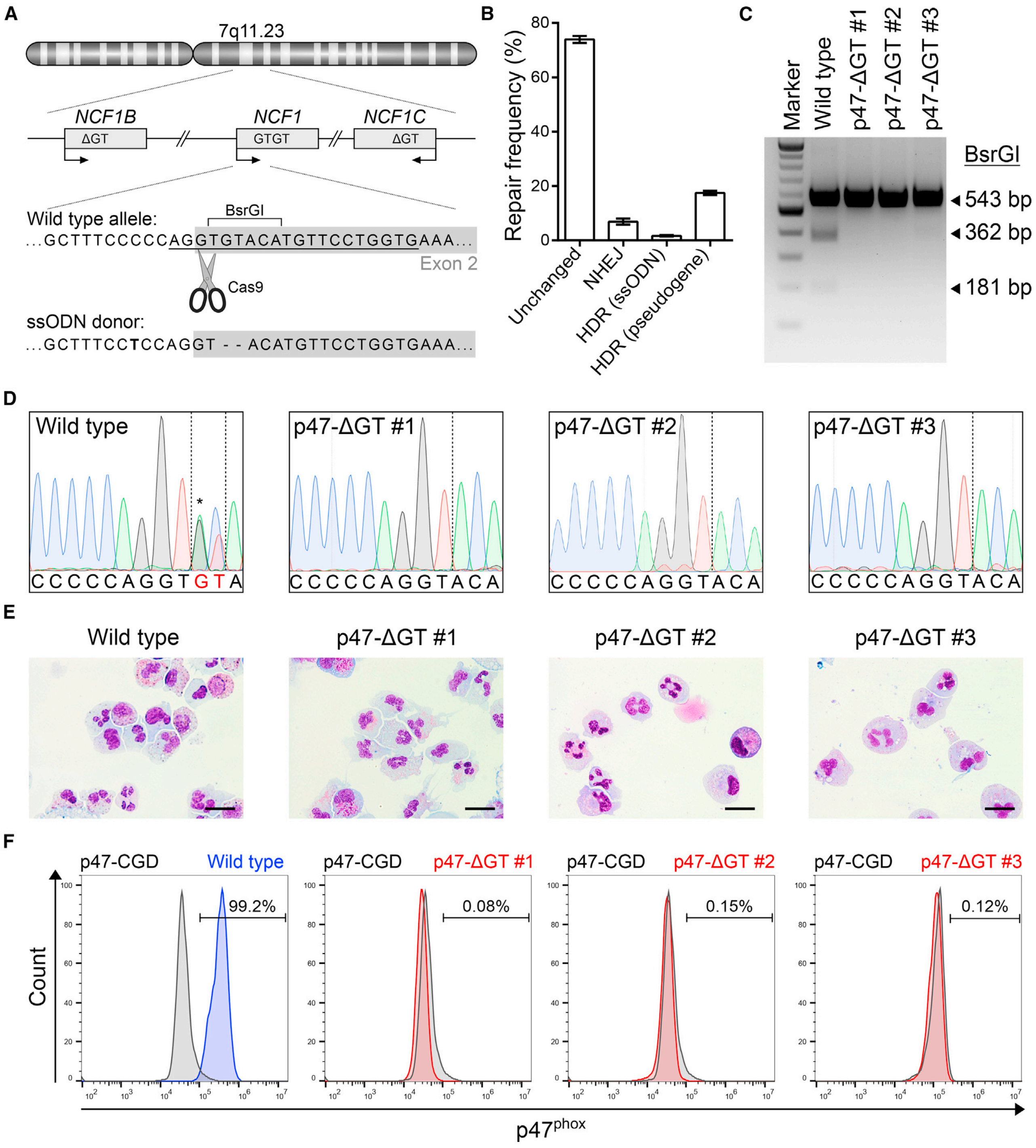

Figure 1. Generation of Patient-like p47-ΔGT iPSCs Using CRISPR/Cas9

(A) Schematic of *NCF1* and its pseudogenes *NCF1B/C* located on chromosome 7. The sgRNA target sequence (underlined) is shown on the wild type allele in the 5′ region of exon 2 (gray box). The donor template (ssODN) carries the ΔGT mutation and a silent point mutation (bold).

(B) Frequency of repaired alleles by non-homologous end-joining (NHEJ) or homology-directed repair (HDR) after Cas9-mediated cleavage. The frequency was determined in sorted bulk populations by next-generation sequencing. The name in parentheses indicates the donor template used (n = 3, mean ± SD).

(legend continued on next page)

p47-ΔGT iPSCs for disease modeling and to develop our gene-correction approach. In contrast to retroviral vectors, the CRISPR/Cas9 system can be applied transiently and allows direct correction of the mutated gene with the advantage that the expression is driven by the endogenous promoter while the original genome architecture remains unaltered. We demonstrate proof of principle that the insertion of a minigene into intron 1 of *NCF1* can correct $p47^{phox}$ deficiency. To specifically target *NCF1*, we chose a position in intron 1 that differs from the pseudogene sequence by an additional three base pairs; thus, the pseudogenes were not targeted by Cas9 and remained intact. Myeloid differentiation of corrected iPSCs revealed restored $p47^{phox}$ expression and functional NADPH oxidase activity measured by reactive oxygen species (ROS) production, formation of neutrophil extracellular traps (NETs), and killing of bacteria.

RESULTS

Generation of Patient-like p47-ΔGT iPSCs Using CRISPR/Cas9

In former CGD iPSC disease models, patient cells were reprogrammed and the disease phenotype was monitored in differentiated hematopoietic cells in comparison with iPSC-derived cells from healthy individuals. However, donor-specific gene expression and genetic variations of iPSC lines may affect the developmental potential (Mills et al., 2013). To investigate the CGD disease phenotype in the same, and therefore comparable, genetic background, we introduced the p47-ΔGT mutation into a well-characterized iPSC line derived from $CD34^+$ blood cells (Ackermann et al., 2014). On-target activity of the specifically designed single guide RNA (sgRNA) p47.ex2 as well as off-target activity in the highly homologous pseudogenes was assessed in an HT1080 reporter cell assay using lentiviral vectors to deliver the all-in-one CRISPR/Cas9 expression cassette together with the on-/off-target reporter constructs (Figure S1A). In this assay, on-target activity was measured via loss of superfolder GFP (sfGFP) fluorescence, and off-target activity in the pseudogenes was analyzed via loss of sfBFP2 fluorescence. Eight days after transduction, p47.ex2 showed an on-target activity of up to 83% with a multiplicity of infection (MOI) of 1, while no significant cleavage was observed in the off-target reporter (Figures S1B–S1D). Subsequently, we proceeded with the transient delivery of CRISPR/Cas9 into iPSCs. Therefore, healthy iPSCs were nucleofected with a single-stranded oligodeoxynucleotide (ssODN) donor template and the all-in-one CRISPR/Cas9 construct, which contains a dTomato fluorescence reporter (Figures 1A and S1A). Thirty-six hours after nucleofection, CRISPR/Cas9-treated iPSCs were sorted for $dTomato^+$ cells and analyzed via next-generation sequencing, which revealed about 25% of genetically modified cells (Figure 1B). The high percentage of unchanged alleles is caused by co-amplification of the pseudogenes during the PCR step, and thus represents an analysis artifact. As no cleavage of the pseudogenes is expected, about 66% of unchanged alleles originate from the pseudogene sequences. Exclusion of these sequences from the calculation to determine the percentage of modified cells led to the estimation that approximately 78% of genetically modified alleles were obtained in *NCF1*, which correlates well with our sgRNA on-target activity of 83% (Figure S1C). In the remaining alleles, 6.9% ± 1.1% had undergone non-homologous end-joining, 1.7% ± 0.3% had used the applied ssODN for homology-directed repair (HDR), and 17.5% ± 0.8% had used one of the pseudogenes as an endogenous donor template for HDR. Sequencing of the top five predicted off-target sites of p47.ex2 revealed no mutations at any of these sites (data not shown). To obtain clonal iPSC lines, we picked and screened single colonies for the desired ΔGT genotype using PCR amplification and a BsrGI restriction digest (Figures 1A and 1C). Three iPSC clones (p47-ΔGT #1, #2, and #3) were identified as correctly modified and further analyzed. Sanger sequencing confirmed the ΔGT genotype (Figure 1D). Analysis of the pluripotency markers SSEA-4 and TRA-1-60 via flow cytometry and of *OCT4*, *NANOG*, and *DNMT3B* by qPCR confirmed that pluripotency was maintained in all iPSC clones after gene editing (Figures S2A and S2B). To evaluate loss of $p47^{phox}$ expression, we differentiated the iPSC clones into granulocytes using an embryoid body-based protocol, which gave rise to neutrophilic granulocytes (Figure 1E). Intracellular antibody staining against $p47^{phox}$ demonstrated complete lack of $p47^{phox}$ expression in differentiated p47-ΔGT cells (Figure 1F).

(C) PCR amplification and BsrGI digest to discriminate correctly modified iPSC clones (BsrGI site removed) from wild type (BsrGI site present).
(D) Sanger sequencing results of wild type iPSCs and p47-ΔGT clones. The wild type sequence shows overlaid sequences of *NCF1* and the pseudogenes due to co-amplification via PCR (asterisk).
(E) Cell morphology of differentiated iPSC-derived granulocytes for wild type and p47-ΔGT cells after Pappenheim staining. Scale bars, 20 μm.
(F) Intracellular staining followed by flow cytometry to detect $p47^{phox}$ expression in wild type and p47-ΔGT granulocytes (gated on p47-CGD granulocytes).

As a control, PBMCs from a p47^{phox}-deficient CGD patient were reprogrammed using a 4-in-1 lentiviral vector expressing OCT4, KLF4, SOX2, and c-MYC (Figure S3A). The overall reprogramming efficiency was very low and only one iPSC clone was obtained (named p47-CGD). Sanger sequencing confirmed the ΔGT genotype (Figure S3B). The clone stained positive for SSEA-4 and TRA-1-60 and expressed the markers *OCT4*, *NANOG*, and *DNMT3B*, demonstrating pluripotency (Figures S3C and S3D). To definitively prove pluripotency, we used the p47-CGD clone in a teratoma formation assay, which gave rise to tissues of all three germ layers (Figure S3E). Finally, the clone was differentiated into granulocytes showing typical cell morphology (Figure S3F) and assessed for p47^{phox} expression. Similar to the Cas9-derived clones, p47-CGD-derived granulocytes lacked p47^{phox} expression (Figure 1F). In summary, we generated patient-like p47-ΔGT iPSCs via CRISPR/Cas9-mediated gene editing, which were genotypically and phenotypically comparable with the patient-derived p47-CGD iPSC line.

Insertion of a Minigene into Intron 1 of *NCF1* Genetically Corrects p47^{phox} Deficiency

The pseudogenes *NCF1B/C* are only distinguishable from *NCF1* by small-nucleotide polymorphisms, smaller insertions, and smaller deletions. Therefore, we used the sgRNA p47.in1 to target a position in intron 1 of *NCF1* that differed from the pseudogenes by three additional base pairs to avoid multiple DSB and off-target insertions of our donor construct into the pseudogenes during CRISPR/Cas9-based gene correction (Figure 2A). Our on-/off-target HT1080 reporter cell assay revealed about 80% on-target activity and lack of cleavage in the pseudogene sequence (Figures S4A–S4D). After insertion of the minigene cassette (pMA.NCF1) and selection of targeted iPSC clones via puromycin resistance, PCR screening revealed 12 out of 55 analyzed iPSC clones to be correctly targeted (28 clones had no detectable insertion at all, despite being selected arguing for insufficient puromycin selection pressure), and 2 out of these 12 clones lacked random insertion (Figure 2B). These corrected iPSC clones (NCF1^{correct} #6 and #10) had two copies of the puromycin resistance gene, which suggested a biallelic targeting (Figure 2C). Sequencing analysis of the region adjacent to the p47.in1 sgRNA target site in *NCF1* and the pseudogenes revealed a lack of the wild type *NCF1* sequence, but presence of both pseudogene sequences in both corrected clones, which provides additional confirmation of a biallelic correction in the *NCF1* locus and of a lack of cleavage activity in the pseudogenes (Figure 2D). Moreover, sequencing of the top five predicted off-target sites for p47.in1 did not reveal any mutations at these sites (data not shown). The corrected iPSC clones also stained positive for the analyzed pluripotency markers after gene correction (Figures S2C and S2D). Finally, the corrected clones were differentiated into granulocytes to analyze restoration of p47^{phox} expression (Figure 2E). As per intracellular p47^{phox} staining, both corrected clones expressed p47^{phox}, although at levels slightly below wild type (Figure 2F). Application of our correction strategy to the patient-derived p47-CGD iPSC clone yielded three clones with biallelic correction out of 33 clones analyzed (Figures S3G and S3H). Differentiated granulocytes had restored p47^{phox} expression (Figures S3I and S3J). For further translation of our correction strategy, we tested the application of CRISPR/Cas9 ribonucleoprotein (RNP) complexes in combination with an adeno-associated virus (AAV) vector to deliver the donor template. In a semi-quantitative PCR, we observed successful gene correction of our iPSCs and achieved similar gene-editing rates compared with our plasmid transfection approach (Figure S4E). Taken together, insertion of a minigene cassette into intron 1 of *NCF1* corrected p47^{phox} deficiency by restoring p47^{phox} expression almost to wild type levels.

Corrected iPSC-Derived Granulocytes Reveal Functional NADPH Oxidase Activity

To analyze the functional capacity of corrected granulocytes, we performed a dihydrorhodamine (DHR) assay. The readout relies on the conversion of DHR to green fluorescent Rho123 in the presence of ROS. Corrected granulocytes were 98.7%–99.0% positive for Rho123 after phorbol 12-myristate 13-acetate (PMA) stimulation, which indicated a strong oxidative burst similar to that measured for wild type granulocytes (Figures 3A and S3K). In contrast, Cas9-created p47-ΔGT granulocytes and patient-derived p47-CGD granulocytes failed to convert DHR to Rho123, confirming the lack of NADPH oxidase activity. Similarly, upon PMA stimulation, wild type and corrected granulocytes produced comparable amounts of ROS over time as measured by oxidation of luminol, while p47-ΔGT and p47-CGD granulocytes were inactive (Figure 3B). Another characteristic of neutrophilic granulocytes is the formation of NETs, which are positively regulated by ROS. NETs consist of chromatin and antimicrobial proteins that are ejected from the cell to trap and kill microbes extracellularly. Upon stimulation of differentiated granulocytes with PMA, the amount of NETs was quantified using fluorescent Sytox green (Figure 3C). The results confirmed that the level of ROS in the corrected cells was sufficient to induce NET formation at efficiencies similar to those of healthy granulocytes, while p47-ΔGT and p47-CGD granulocytes were unable to form NETs. In conclusion, we demonstrated that restored p47^{phox} expression in corrected granulocytes resulted in functional NADPH oxidase activity, ROS production, and NET formation.

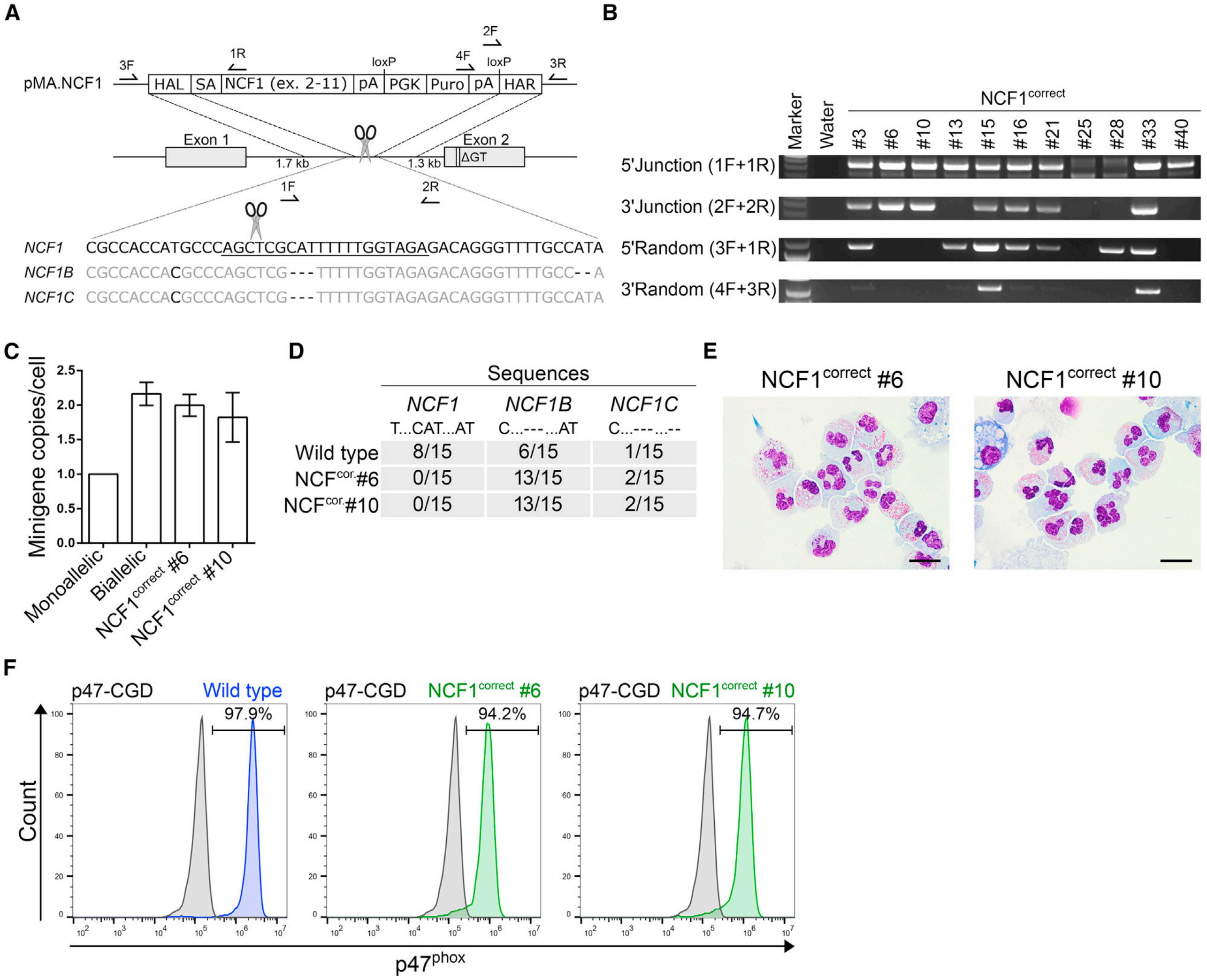

Figure 2. Genetic Correction of p47-ΔGT iPSCs by Targeted Insertion of a Minigene into Intron 1 of *NCF1*

(A) Schematic of gene editing and correction strategy. The sgRNA target sequence (underlined) differs from the pseudogene sequence by three additional nucleotides. Arrows indicate positions of primers used for genotyping. F, forward primer; R, reverse primer; HAL, homology arm left; SA, splice acceptor site; NCF1, cDNA encoding the *NCF1* gene excluding exon 1; pA, polyadenylation signal; PGK, phosphoglycerate kinase promoter; Puro, puromycin resistance gene; HAR, homology arm right.

(B) PCR-based genotyping. Applied primers were used as indicated, and their binding sites are depicted in (A).

(C) Determination of the minigene copy number normalized to the *PTBP2* gene via qPCR (n = 3, mean ± SD, technical replicates).

(D) Sequencing analysis of the region adjacent to the p47.in1 target site in *NCF1* and the pseudogenes. The PCR fragment was subcloned and single clones were analyzed by Sanger sequencing. Sequences were identified as *NCF1*, *NCF1B*, or *NCF1C* based on specific point mutations and deletions as indicated.

(E) Cell morphology of corrected iPSC-derived granulocytes after Pappenheim staining. Scale bars, 20 μm.

(F) Intracellular staining followed by flow cytometry to detect $p47^{phox}$ expression in wild type and corrected granulocytes (gated on p47-CGD granulocytes).

Corrected iPSC-Derived Macrophages Kill Phagocytosed Bacteria

In addition to granulocytes, the function of macrophages is also compromised in CGD patients. To investigate the benefit of the genetic correction, we differentiated our iPSC clones into macrophages. The cells showed typical macrophage morphology in Pappenheim staining (Figure 4A). To demonstrate that the differentiated macrophages were able to kill phagocytosed bacteria, we infected macrophage cultures with sfGFP-labeled *Escherichia coli* and analyzed the amount of living bacteria present inside the macrophages after 24 h of incubation. The bacterial

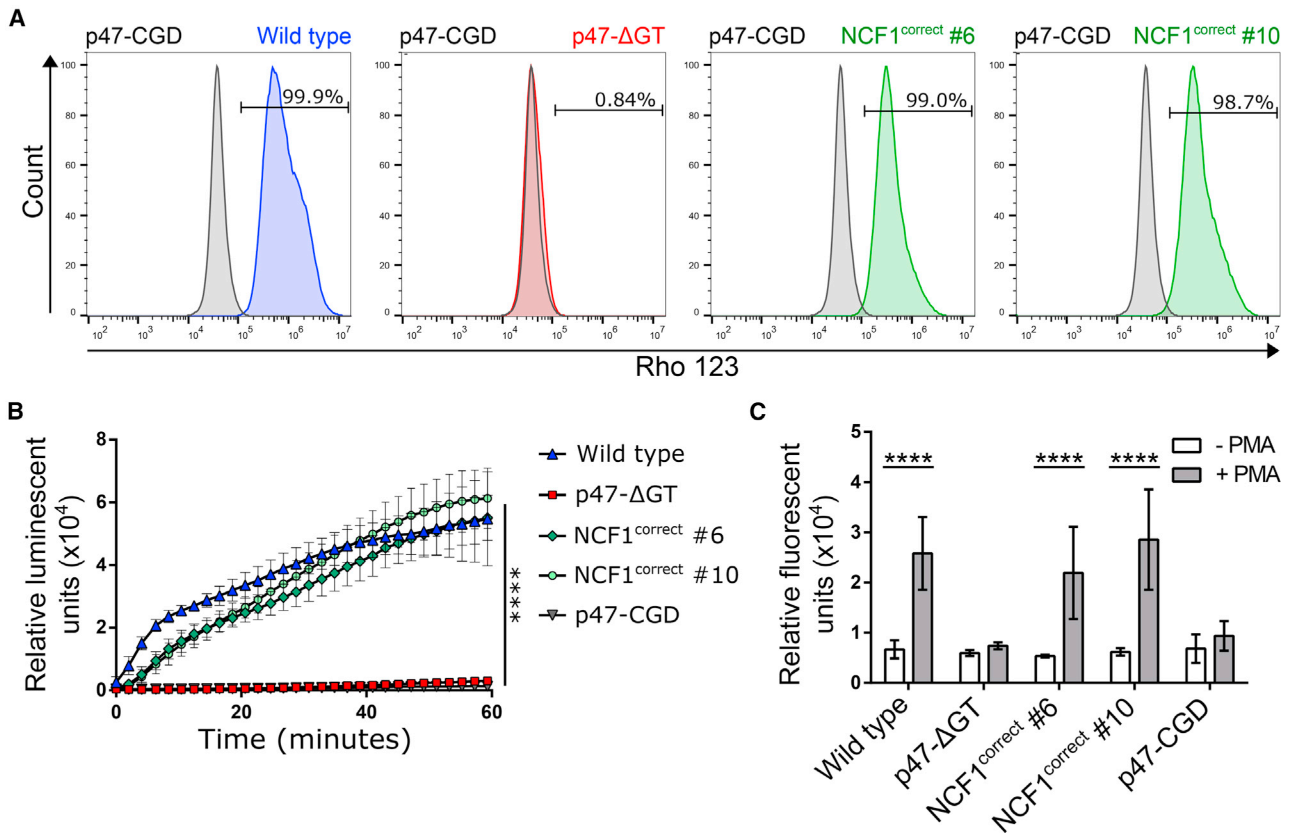

Figure 3. Functional Characterization of Corrected iPSC-Derived Granulocytes

(A) DHR assay. After PMA stimulation, DHR is converted in the presence of ROS to green fluorescent Rho123 and assessed by flow cytometry (gated on stimulated p47-CGD granulocytes).

(B) Chemiluminescent ROS assay. ROS production was measured in the presence of luminol over time in granulocytes after PMA stimulation (n = 4–7 pooled from three independent experiments; mean ± SD, one-way ANOVA; ****$p \leq 0.0001$).

(C) NET formation assay. NET formation was quantified by Sytox green staining 2 h after PMA stimulation and compared with unstimulated granulocytes (n = 3–9 pooled from three independent experiments; mean ± SD, two-way ANOVA; ****$p \leq 0.0001$). Significance is only shown for stimulation.

load was determined by plating cell lysates and scoring of colony-forming units. Over 90% of all differentiated macrophages were able to phagocytose sfGFP-positive *E. coli* (Figures 4B and 4C). The killing assay clearly demonstrated that wild type and corrected macrophages were able to kill phagocytosed *E. coli* via their respiratory burst, whereas p47-ΔGT and p47-CGD macrophages retained significantly more living bacteria inside their phagosomes (Figure 4D). Furthermore, the NADPH oxidase activity appeared to be necessary for macrophages to kill phagocytosed bacteria and thus to enable the clearance of infections.

DISCUSSION

Targeted repair of the p47-ΔGT mutation in CGD cells is a challenging task for gene editing by CRISPR/Cas9 due to the presence of two highly homologous pseudogenes, which also carry the most frequent disease-causing mutation (ΔGT) (Roesler et al., 2000). The high sequence homology can cause unwanted off-target DSB in the pseudogenes by the Cas9. The repair of multiple breaks can then lead to larger deletions, inversions, and translocations, and thus, potentially, to chromosomal instability. In this study, we used CRISPR/Cas9 to generate a p47-ΔGT iPSC line for subsequent gene therapy development. Upon introduction of the ΔGT mutation into exon 2 of healthy iPSCs, we observed that CRISPR/Cas9 exhibited a 10-fold higher preference for using the pseudogenes as endogenous donors compared with the exogenously applied donor template. The resultant reintroduction of the pseudogene sequence into the target site via recombination could reduce the overall gene-correction frequency when applying gene editing in autologous HSCs to treat $p47^{phox}$ deficiency.

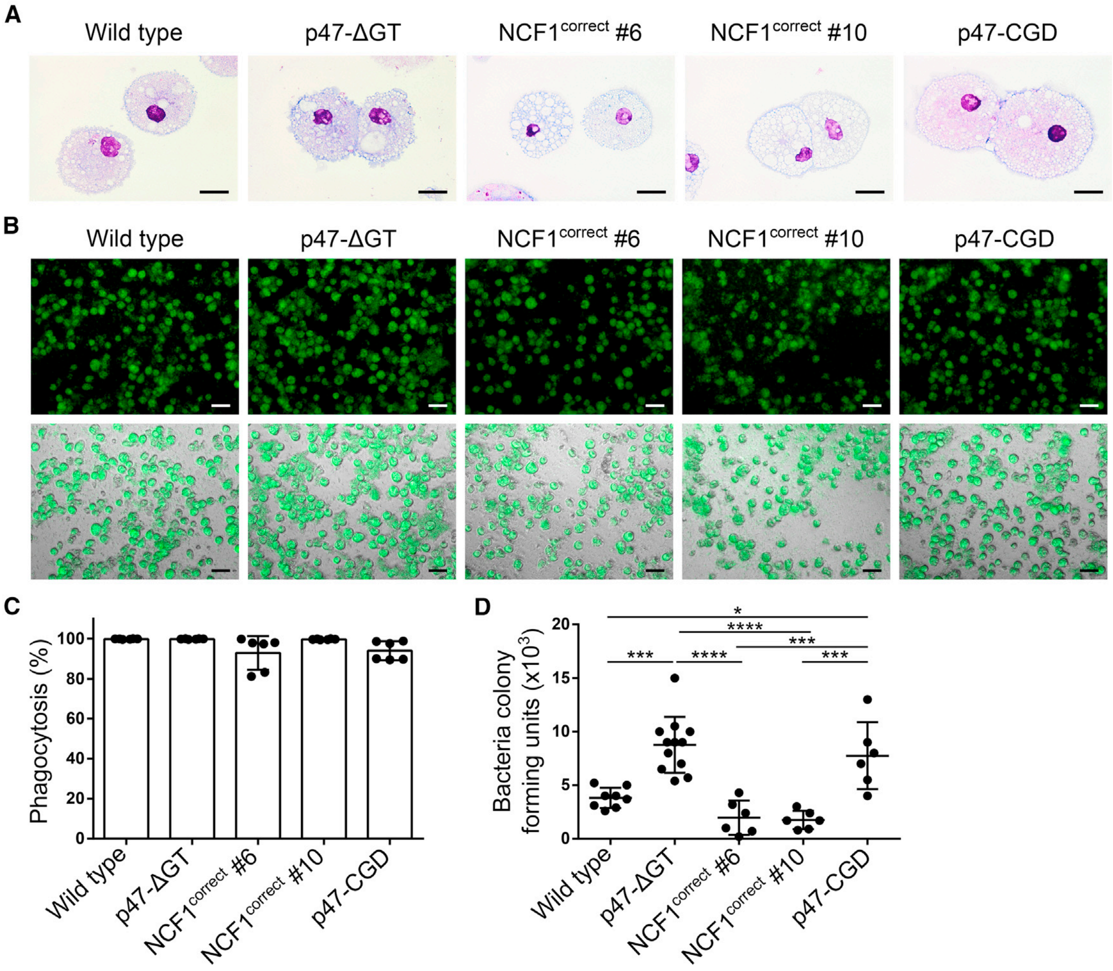

Figure 4. Corrected iPSC-Derived Macrophages Kill Phagocytosed Bacteria

(A) Cell morphology of iPSC-derived macrophages after Pappenheim staining. Scale bars, 20 μm.

(B–D) *E. coli* killing assay. (B) Macrophages are infected with sfGFP-labeled *E. coli* at an MOI of 1. Shown are microscopic pictures of macrophages that phagocytosed bacteria. Top panel: sfGFP; bottom panel: bright-field + sfGFP (scale bars, 50 μm). (C) Phagocytosis rate of total macrophages measured 6 h after infection with sfGFP-labeled *E. coli* by flow cytometry (n = 6 pooled from three independent experiments; mean ± SD, one-way ANOVA—no significance). (D) Colony-forming units of *E. coli* bacteria isolated from cell lysates of infected macrophages 24 h after infection (n = 6–12 pooled from three independent experiments; mean ± SD, one-way ANOVA; *$p \leq 0.05$, ***$p \leq 0.001$, ****$p \leq 0.0001$).

Besides the fact that the pseudogenes carry the ΔGT mutation and serve as an endogenous donor template, the functional role of the pseudogenes is poorly understood. Whether the pseudogenes might act, for example, as non-coding RNAs that affect the transcriptome remains to be elucidated. Still contradictory, some studies indicate that certain pseudogene transcripts serve as a modulator to balance superoxide production (Xu et al., 2015) and that there might be a correlation of the ΔGT/GTGT ratio with diseases, such as inflammatory bowel disease or the onset of autoimmunity (Greve et al., 2008; Harbord et al., 2003). Thus, we decided to develop a CRISPR/Cas9 gene-correction approach for p47^{phox} deficiency that avoids cleavage of the pseudogene sequences. We identified a unique sgRNA target sequence in intron 1 of *NCF1* that allows specific insertion of our *NCF1* minigene and, thus, correction of all downstream *NCF1* mutations. We demonstrated that the corrected iPSC-derived granulocytes have restored p47^{phox} expression similar to wild type levels. Moreover, the insertion did not result in the disruption of any enhancer elements in intron 1 needed for expression as shown by comparable p47^{phox} expression levels. In a similar approach, Sweeney et al. (2017) inserted a minigene into exon 1 of *CYBB* (encoding gp91^{phox}) to treat X-CGD. In their study, however, the insertion did not result in the expression of the therapeutic gp91^{phox}. This experiment

indicates that there may be non-coding elements in intron 1 of *CYBB* necessary for expression from the *CYBB* promoter.

To continue, we explored the use of iPSCs to recapitulate the pathogenesis of p47-CGD and develop a gene therapy strategy. The major advantages of iPSCs are that they represent an unlimited source of stem cells. Furthermore, they offer the possibility of detailed clonal analysis and selection prior to expansion and therapeutic application. Our functional assays demonstrated that the corrected iPSC-derived granulocytes and macrophages display all the features necessary to fight infections: sufficient ROS levels, induction of NET formation, and the ability to kill bacteria. Some CGD patients, who suffer from severe infections, benefit from granulocyte transfusions. However, this treatment is complicated by alloimmunization and may be detrimental for subsequent allogeneic HSCT (Connelly et al., 2018). Beyond their value for disease modeling, corrected iPSC-derived neutrophils and macrophages could be used as alternative autologous cell sources to combat intractable infections in CGD patients. In two different studies, iPSC-derived neutrophils or macrophages were successfully applied to treat bacterial infections of the peritoneum or the lung (Trump et al., 2019; Ackermann et al., 2018). Recently, the application of CRISPR-RNPs in combination with an AAV6 to deliver the donor template has yielded high HDR rates (60%–70%) during gene editing of HSCs (Vakulskas et al., 2018). We obtained preliminary data that we can induce HDR in our iPSC disease model via RNP/AAV delivery, and we are confident that the technique is transferable for application in patient-derived HSCs. However, it remains to be tested whether these tools could result in a sufficient amount of corrected granulocytes (~5%–20%) to protect patients from severe infections (Marciano et al., 2017b).

Our study provides proof of concept for a successful CRISPR/Cas9-mediated gene correction of $p47^{phox}$ deficiency. The functionality of the effector cells was restored, which provides the basis for gene and cell therapy treatments, such as iPSC-derived granulocyte transfusions. Much progress has been made in the last years to develop safer therapies for CGD patients, including gene therapy. This progress converted CGD from a formerly fatal disease of childhood into a disease with elaborated therapy options and a longer life expectancy.

EXPERIMENTAL PROCEDURES

Gene Editing of iPSCs

Genetic modification was performed via nucleofection of CRISPR/Cas9 components using the Amaxa Nucleofector II and the mouse ES Cell Nucleofection Kit according to the manufacturer (both Lonza). For disease modeling, 2×10^6 healthy iPSCs were nucleofected with 2.5 μg of CRISPR/Cas9 plasmid (sgRNA p47.ex2 + Cas9.2A.dTomato) and 100 pmol of a 120-bp ssODN as donor template. After fluorescence-activated cell sorting (FACSAria Fusion, Becton Dickinson) for $dTomato^+$ iPSCs, colonies were expanded from single cells and analyzed (see Supplemental Information).

For genetic correction, 2×10^6 $p47^{phox}$-deficient iPSCs (p47-ΔGT #1) were nucleofected in the presence of 2.5 μg of CRISPR/Cas9 plasmid (sgRNA p47.in1 + Cas9.2A.dTomato) and 2.5 μg of the homology donor pMA.NCF1. Two days after nucleofection, iPSCs were selected using 0.2 μg/mL puromycin (InvivoGen) on multiresistant mouse embryonic feeder cells (strain DR4) kindly provided by T. Cantz (Hannover Medical School). Single iPSC colonies were analyzed for correct targeting.

Further experimental procedures are provided in Supplemental Experimental Procedures.

SUPPLEMENTAL INFORMATION

Supplemental Information can be found online at https://doi.org/10.1016/j.stemcr.2019.08.008.

AUTHOR CONTRIBUTIONS

D.K.: conception and design, data collection, analysis and interpretation, manuscript writing. E.C., F.P., and J.D.: performed experiments. A. Selich: next-generation sequencing data analysis. R.E.S.: provided patient material. H.B.: provided vector material, conceptual advice, discussed results. J.W.S., D.H.: provided conceptual advice, discussed results. A.J.T.: discussed results, data analysis, data interpretation. A. Schambach: conception and design, financial support, data analysis, data interpretation. All authors contributed to manuscript preparation and approved the final version of the manuscript.

ACKNOWLEDGMENTS

This work was supported by grants from the German Research Foundation (SFB738 (C9) and REBIRTH Cluster of Excellence (EXC 62/2)), the Federal Ministry of Education and Research (Pidnet/FK2016M1517F; iMACnet/01EK1602A), and the German Academic Scholarship Foundation. In addition, this project has received funding from the European Union's Horizon 2020 research and innovation programme under grant agreement nos. 666908 and 755170. A.J.T. is supported by the Wellcome Trust (104807/Z/14/Z) and the NIHR Biomedical Research Centre at Great Ormond Street Hospital for Children NHS Foundation Trust and University College London. We thank Michael Morgan (Hannover Medical School, Germany) for proofreading the manuscript, and Manuel Grez for helpful suggestions and discussion of the project.

Received: January 11, 2019
Revised: August 17, 2019
Accepted: August 18, 2019
Published: September 19, 2019

REFERENCES

Ackermann, M., Kempf, H., Hetzel, M., Hesse, C., Hashtchin, A.R., Brinkert, K., Schott, J.W., Haake, K., Kühnel, M.P., Glage, S., et al.

(2018). Bioreactor-based mass production of human iPSC-derived macrophages enables immunotherapies against bacterial airway infections. Nat. Commun. *9*, 5088–6001.

Ackermann, M., Lachmann, N., Hartung, S., Eggenschwiler, R., Pfaff, N., Happle, C., Mucci, A., Göhring, G., Niemann, H., Hansen, G., et al. (2014). Promoter and lineage independent anti-silencing activity of the A2 ubiquitous chromatin opening element for optimized human pluripotent stem cell-based gene therapy. Biomaterials *35*, 1531–1542.

Connelly, J.A., Marsh, R., Parikh, S., and Talano, J. (2018). Allogeneic hematopoietic cell transplantation for chronic granulomatous disease: controversies and state of the art. J. Pediatr. Infect. Dis. Soc. *7*, 31–39.

Greve, B., Hoffmann, P., Vonthein, R., Kun, J., Lell, B., Mycko, M.P., Selmaj, K.W., Berger, K., Weissert, R., and Kremsner, P.G. (2008). NCF1 gene and pseudogene pattern: association with parasitic infection and autoimmunity. Malar. J. *7*, 251–257.

Güngör, T., Teira, P., Slatter, M., Stussi, G., Stepensky, P., Moshous, D., Vermont, C., Ahmad, I., Shaw, P.J., Telles da Cunha, J.M., et al. (2014). Reduced-intensity conditioning and HLA-matched haemopoietic stem-cell transplantation in patients with chronic granulomatous disease: a prospective multicentre study. Lancet *383*, 436–448.

Harbord, M., Hankin, A., Bloom, S., and Mitchison, H. (2003). Association between p47phox pseudogenes and inflammatory bowel disease. Blood *101*, 3337.

Holland, S.M. (2010). Chronic granulomatous disease. Clin. Rev. Allergy Immunol. *38*, 3–10.

Marciano, B.E., Allen, E.S., Conry-Cantilena, C., Kristosturyan, E., Klein, H.G., Fleisher, T.A., Holland, S.M., Malech, H.L., and Rosenzweig, S.D. (2017a). Granulocyte transfusions in patients with chronic granulomatous disease and refractory infections: the NIH experience. J. Allergy Clin. Immunol. *140*, 622–625.

Marciano, B.E., Zerbe, C.S., Falcone, E.L., Ding, L., DeRavin, S.S., Daub, J., Kreuzburg, S., Yockey, L., Hunsberger, S., Foruraghi, L., et al. (2017b). X-linked carriers of chronic granulomatous disease: illness, lyonization and stability. J. Allergy Clin. Immunol. *141*, 365–371.

Merling, R.K., Kuhns, D.B., Sweeney, C.L., Wu, X., Burkett, S., Chu, J., Lee, J., Koontz, S., Di Pasquale, G., Afione, S.A., et al. (2017). Gene-edited pseudogene resurrection corrects p47phox-deficient chronic granulomatous disease. Blood Adv. *1*, 270–278.

Mills, J.A., Wang, K., Paluru, P., Ying, L., Lu, L., Galvão, A.M., Xu, D., Yao, Y., Sullivan, S.K., Sullivan, L.M., et al. (2013). Clonal genetic and hematopoietic heterogeneity among human-induced pluripotent stem cell lines. Blood *122*, 2047–2051.

Ott, M.G., Schmidt, M., Schwarzwaelder, K., Stein, S., Siler, U., Koehl, U., Glimm, H., Kühlcke, K., Schilz, A., Kunkel, H., et al. (2006). Correction of X-linked chronic granulomatous disease by gene therapy, augmented by insertional activation of MDS1-EVI1, PRDM16 or SETBP1. Nat. Med. *12*, 401–409.

Reeves, E.P., Lu, H., Jacobs, H.L., Messina, C.G.M., Bolsover, S., Gabella, G., Potma, E.O., Warley, A., Roes, J., and Segal, A.W. (2002). Killing activity of neutrophils is mediated through activation of proteases by K^+ flux. Nature *416*, 291–297.

Roesler, J., Curnutte, J.T., Rae, J., Barrett, D., Patino, P., Chanock, S.J., and Goerlach, A. (2000). Recombination events between the p47-phox gene and its highly homologous pseudogenes are the main cause of autosomal recessive chronic granulomatous disease. Blood *95*, 2150–2156.

Roos, D. (1994). The genetic basis of chronic granulomatous disease. Immunol. Rev. *138*, 121–157.

Roos, D., Kuhns, D.B., Maddalena, A., Bustamante, J., Kannengiesser, C., de Boer, M., van Leeuwen, K., Köker, M.Y., Wolach, B., Roesler, J., et al. (2010). Hematologically important mutations: the autosomal recessive forms of chronic granulomatous disease (second update). Blood Cells Mol. Dis. *44*, 291–299.

Seger, R.A. (2008). Modern management of chronic granulomatous disease. Br. J. Haematol. *140*, 255–266.

Sweeney, C.L., Zou, J., Choi, U., Merling, R.K., Liu, A., Bodansky, A., Burkett, S., Kim, J., De Ravin, S.S., and Malech, H.L. (2017). Targeted repair of CYBB in X-CGD iPSCs requires retention of intronic sequences for expression and functional correction. Mol. Ther. *25*, 321–330.

Trump, L.R., Nayak, R.C., Singh, A.K., Emberesh, S., Wellendorf, A.M., Lutzko, C.M., and Cancelas, J.A. (2019). Neutrophils derived from genetically modified human induced pluripotent stem cells circulate and phagocytose bacteria in vivo. Stem Cells Transl. Med. *8*, 557–567.

Vakulskas, C.A., Dever, D.P., Rettig, G.R., Turk, R., Jacobi, A.M., Collingwood, M.A., Bode, N.M., McNeill, M.S., Yan, S., Camarena, J., et al. (2018). A high-fidelity Cas9 mutant delivered as a ribonucleoprotein complex enables efficient gene editing in human hematopoietic stem and progenitor cells. Nat. Med. *24*, 1216–1224.

Xu, W., Ma, L., Li, W., Brunson, T.A., Tian, X., Richards, J., Li, Q., Bythwood, T., Yuan, Z., and Song, Q. (2015). Functional pseudogenes inhibit the superoxide production. Precis. Med. *1*, 1–9.

Stem Cell Reports, Volume *13*

Supplemental Information

Targeted Repair of p47-CGD in iPSCs by CRISPR/Cas9: Functional Correction without Cleavage in the Highly Homologous Pseudogenes

Denise Klatt, Erica Cheng, Friederike Philipp, Anton Selich, Julia Dahlke, Reinhold E. Schmidt, Juliane W. Schott, Hildegard Büning, Dirk Hoffmann, Adrian J. Thrasher, and Axel Schambach

Inventory of Supplemental Information

- Figure S1. Evaluation of p47.ex2 sgRNA on-target activity. Related to Figure 1.
- Figure S2. Gene editing of iPSCs does not alter pluripotency marker expression. Related to Figures 1 and 2.
- Figure S3. Reprogramming of patient-derived PBMCs for p47-CGD disease modeling and correction. Related to Figure 1.
- Figure S4. Evaluation of p47.in1 sgRNA on-target activity. Related to Figure 2.
- Supplemental Experimental Procedures
- Table S1. Primers/oligodeoxynucleotides used in this study. Related to Figure 1, Figure 2, Figure S2, Figure S3 and Figure S4.
- Supplemental References

Figure S1. Evaluation of p47.ex2 sgRNA on-target activity. Related to Figure 1.

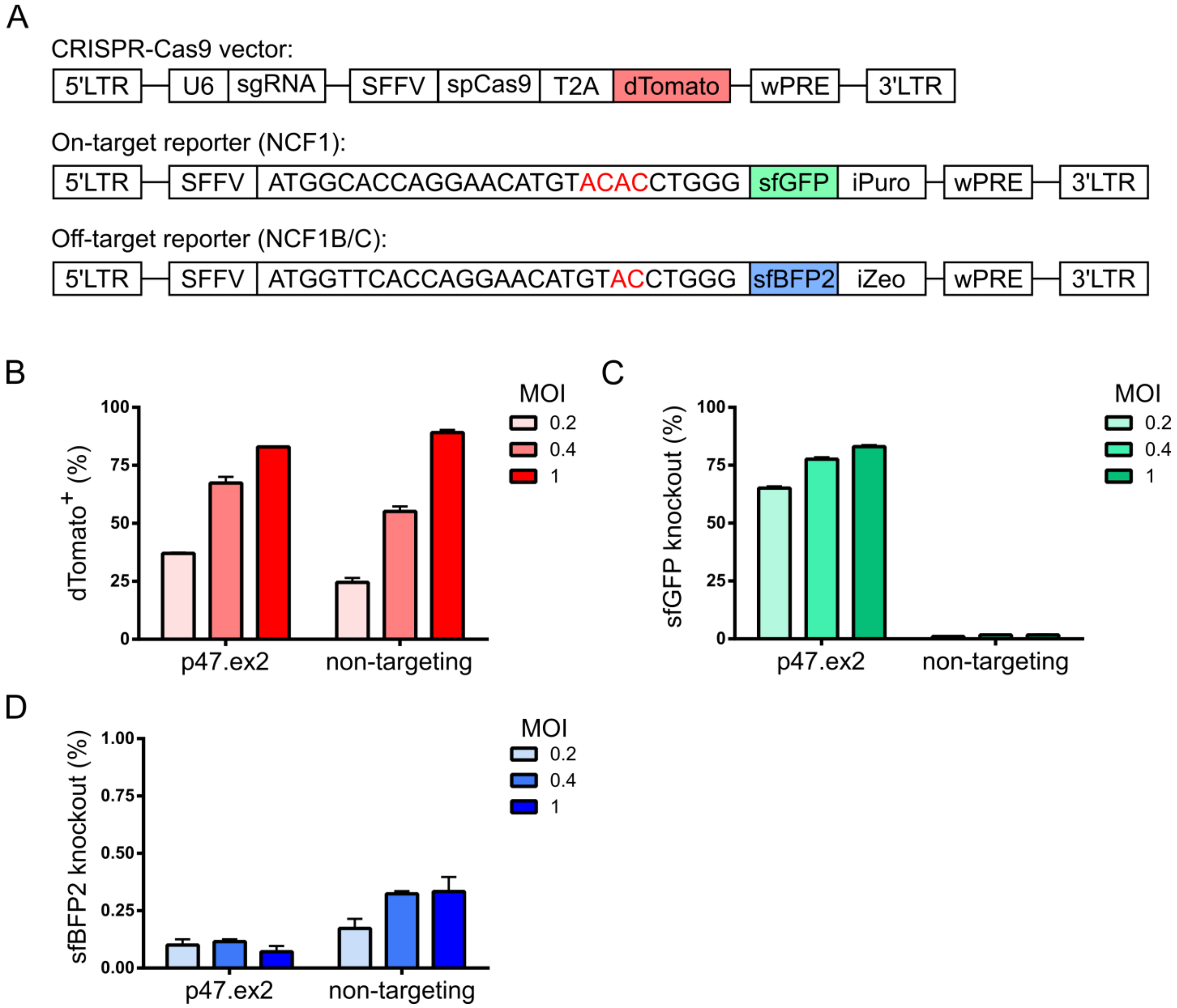

Figure S1. Evaluation of p47.ex2 sgRNA on-target activity. Related to Figure 1. **(A)** Lentiviral vectors used to evaluate sgRNA on-target activity in a HT1080 reporter cell line. The ΔGT mutation is indicated in red (antisense strand is shown). 5'LTR, 5' long terminal repeat; U6, human U6 promoter; sgRNA, single-guide RNA; SFFV, spleen focus-forming virus promoter; spCas9, *Streptococcus pyogenes*-derived Cas9; T2A, peptide cleavage site; dTomato, a red fluorescent protein; wPRE, woodchuck posttranscriptional regulatory element; 3'LTR, 3' long terminal repeat; sfGFP, super-folder green fluorescent protein; iPuro, internal ribosomal entry site (IRES, abbreviated: i) puromycin resistance gene; sfBFP2, super-folder blue fluorescent protein 2; Zeo, zeocin. **(B)** HT1080 dual on-/off-target reporter cells were transduced with the CRISPR/Cas9 vector at different MOI. Shown is the transduction rate measured as dTomato$^+$ cells in the total HT1080 cell population by flow cytometry (n = 3; mean ± SD). MOI, multiplicity of infection; p47.ex2, sgRNA targeting exon 2 of *NCF1*; non-targeting, non-targeting sgRNA as control. **(C)** On-target cleavage activity. Loss of sfGFP fluorescence is a surrogate marker for Cas9-mediated cleavage of the on-target reporter and was measured as sfGFP$^-$ cells in the dTomato$^+$ population by flow cytometry (n = 3; mean ± SD). **(D)** Off-target cleavage activity. Loss of sfBFP2 fluorescence is a surrogate marker for Cas9-mediated cleavage of the off-target reporter and was measured as sfBFP2$^-$ cells in the dTomato$^+$ population (n = 3; mean ± SD).

Figure S2. Gene editing of iPSCs does not alter pluripotency marker expression. Related to Figure 1 and Figure 2.

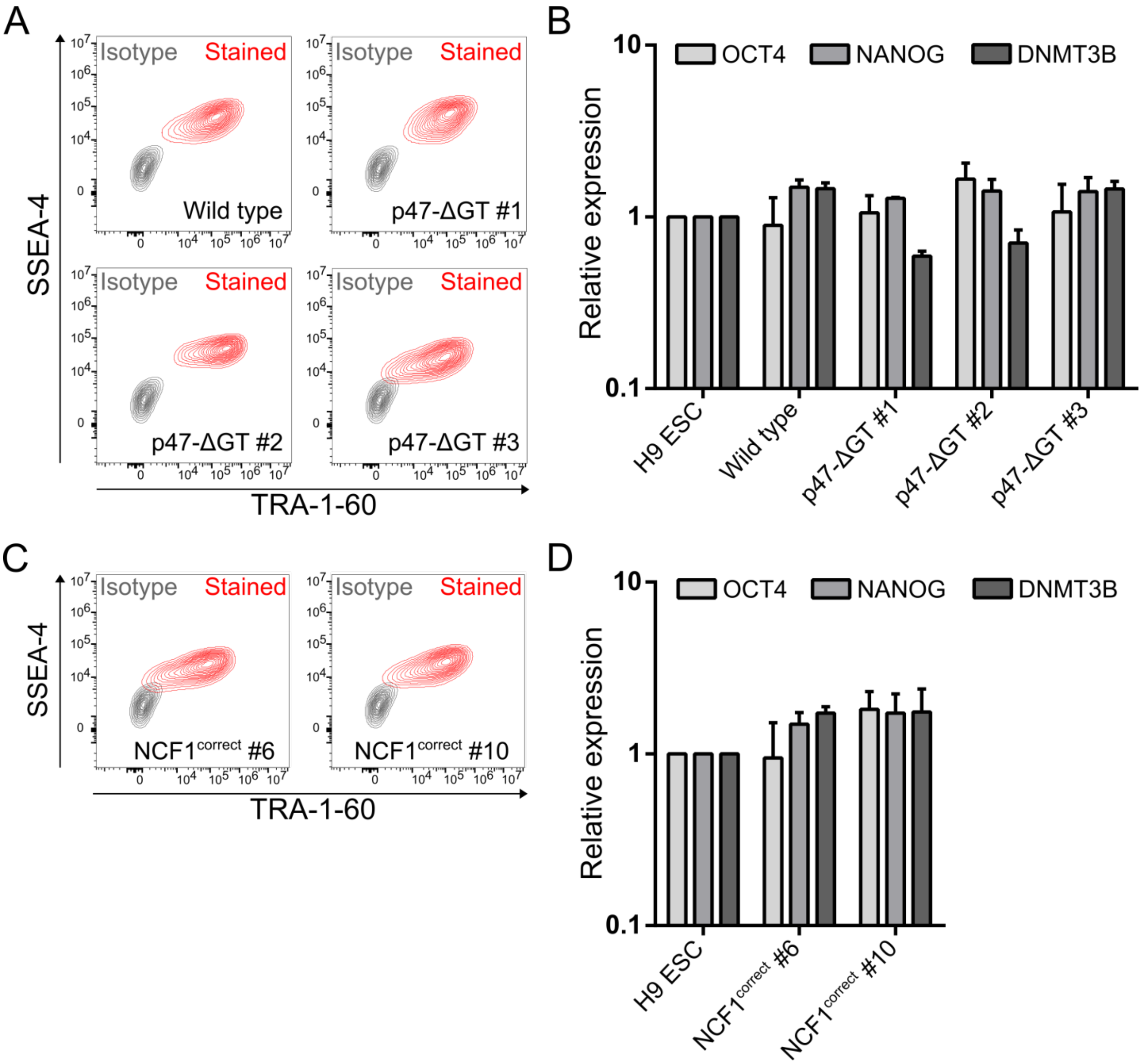

Figure S2. Gene editing of iPSCs does not alter pluripotency marker expression. Related to Figures 1 and 2. **(A, C)** Expression of the pluripotency markers SSEA-4 and TRA-1-60 measured in viable iPSCs by flow cytometry. **(B, D)** Quantitative PCR analysis assessing mRNA expression levels of *OCT4*, *NANOG* and *DNMT3B* in iPSCs normalized to *β-ACTIN* and relative to H9 embryonic stem cells (ESC).

Figure S3. Reprogramming of patient-derived PBMCs for p47-CGD disease modeling and correction. Related to Figure 1.

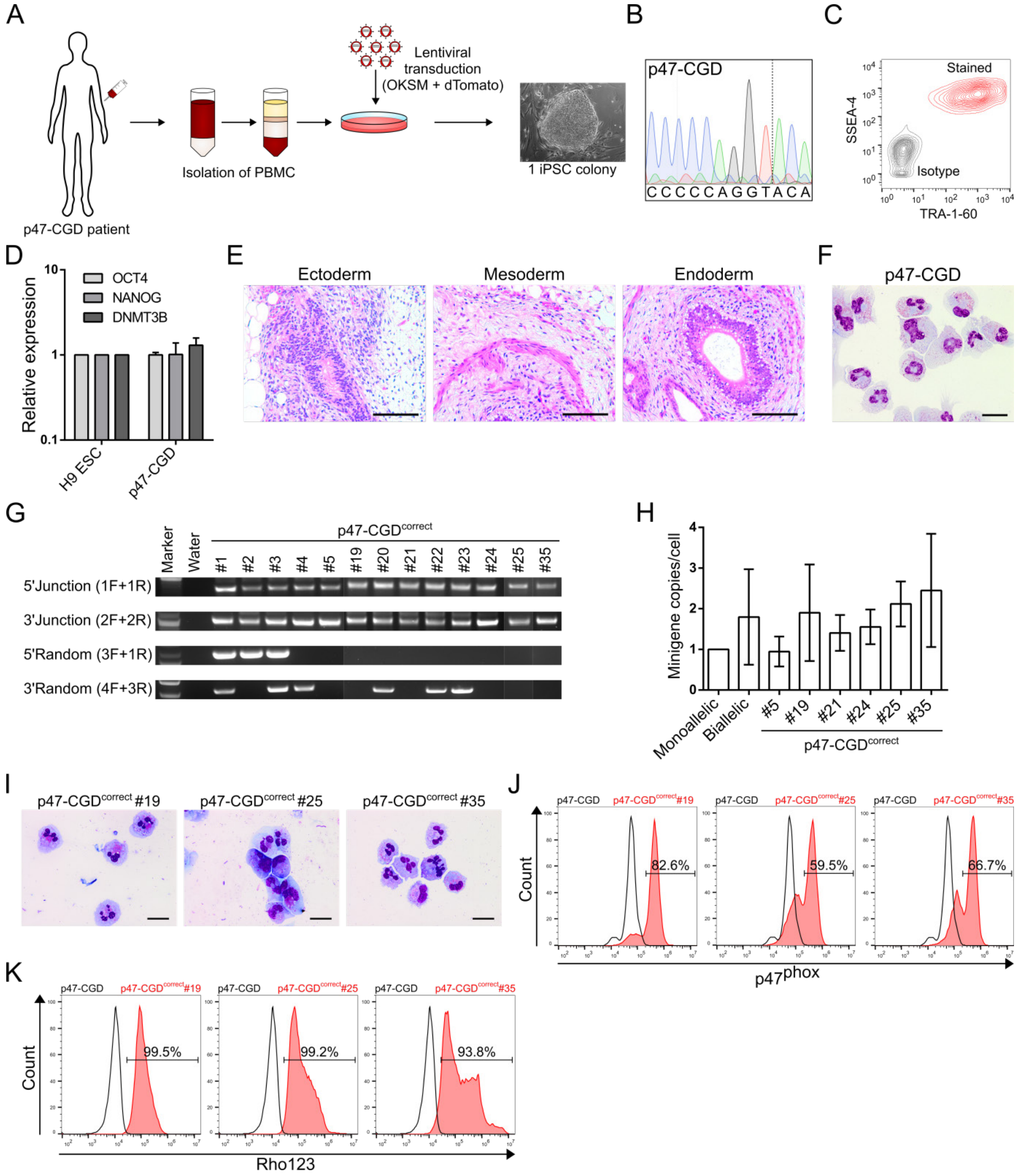

Figure S3. Reprogramming of patient-derived PBMCs for p47-CGD disease modeling and correction. Related to Figure 1. **(A)** Scheme showing reprogramming of p47-CGD patient-derived PBMCs. Isolated PBMCs were transduced with a lentiviral vector expressing the transcription factors OCT4, KLF4, SOX2 and c-MYC. Morphology of the obtained p47-CGD iPSC clone is shown. **(B)** Sanger sequencing analysis of the p47-CGD iPSC clone confirmed the presence of the ΔGT mutation. **(C)** Flow cytometric analysis of the pluripotency markers SSEA-4 and TRA-1-60 in viable iPSCs. **(D)** Quantitative PCR analysis assessing mRNA expression levels of *OCT4*, *NANOG* and *DNMT3B* in iPSCs normalized to β-*ACTIN* and relative to H9 ESCs. **(E)** Teratoma formation assay. Shown are sections from a p47-CGD iPSC-derived teratoma stained with hematoxylin eosin that represent tissues of all three germ layers (scale bar = 100 µm). **(F)** Cell morphology of p47-CGD iPSC-derived granulocytes after Pappenheim staining (scale bar = 20 µm). **(G)** PCR-based genotyping. Applied primers were used as depicted in Figure 2A. **(H)** Determination of the minigene copy number normalized to the *PTBP2* gene via qPCR (n=3, mean ± SD, technical replicates). **(I)** Cell morphology of corrected p47-CGD iPSC-derived granulocytes after Pappenheim staining (scale bar = 20 µm). **(J)** Intracellular staining followed by flow cytometry to detect $p47^{phox}$ expression in corrected p47-CGD iPSC-derived granulocytes (gated on p47-CGD granulocytes). **(K)** DHR assay of corrected p47-CGD iPSC-derived granulocytes (gated on p47-CGD granulocytes).

Figure S4. Evaluation of p47.in1 sgRNA on-target activity. Related to Figure 2.

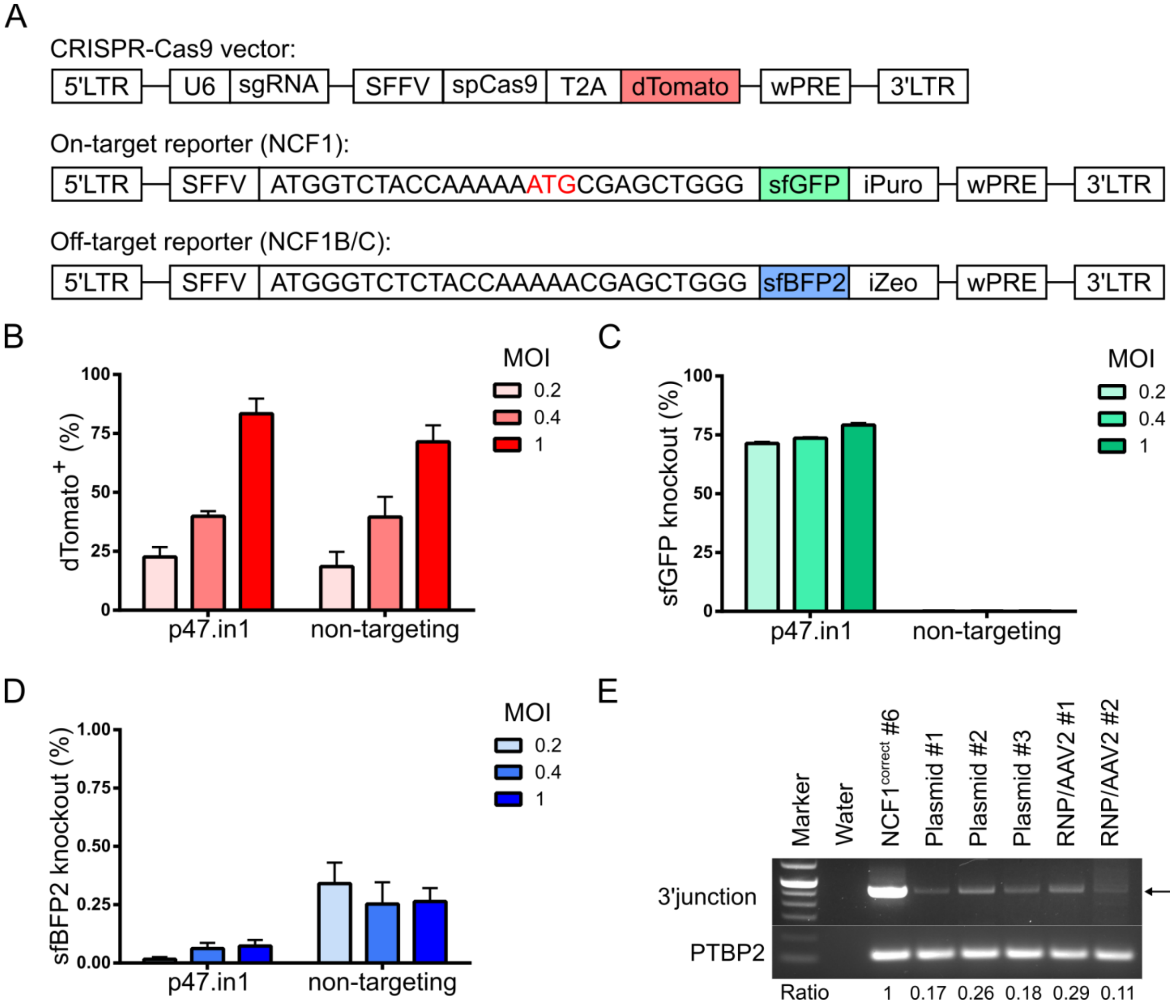

Figure S4. Evaluation of p47.in1 sgRNA on-target activity. Related to Figure 2. **(A)** Lentiviral vectors used to evaluate sgRNA on-target activity in an HT1080 reporter cell line. The three additional nucleotides are shown in red. 5’LTR, 5’ long terminal repeat; U6, human U6 promoter; sgRNA, single-guide RNA; SFFV, spleen focus-forming virus promoter; spCas9, *Streptococcus pyogenes*-derived Cas9; T2A, peptide cleavage site; dTomato, a red fluorescent protein; wPRE, woodchuck posttranscriptional regulatory element; 3’LTR, 3’ long terminal repeat; sfGFP, super-folder green fluorescent protein; iPuro, internal ribosomal entry site (IRES, short: i) and puromycin resistance gene; sfBFP2, super-folder blue fluorescent protein 2; Zeo, zeocin. **(B)** HT1080 dual on-/off-target reporter cells were transduced with the CRISPR/Cas9 vector at different MOI. Shown is the transduction rate measured as dTomato^{+} cells in the total HT1080 cell population by flow cytometry (n = 3; mean ± SD). MOI, multiplicity of infection; p47.in1, sgRNA targeting intron 1 of *NCF1*; non-targeting, non-targeting sgRNA as control. **(C)** On-target cleavage activity. Loss of sfGFP fluorescence is a surrogate marker for Cas9-mediated cleavage of the on-target reporter and was measured as sfGFP^{-} cells in the dTomato^{+} population by flow cytometry (n = 3; mean ± SD). **(D)** Off-target cleavage activity. Loss of sfBFP2 fluorescence is a surrogate marker for Cas9-mediated cleavage of the off-target reporter and was measured as sfBFP2^{-} cells in the dTomato^{+} population (n = 3; mean ± SD). **(E)** Semi-quantitative PCR to assess the gene editing efficiency of the sgRNA p47.in1 using plasmid transfection and RNP/AAV2 delivery. The ratio represents the relative intensity of the 3’junction divided by PTBP2 and normalized to NCF1^{correct} #6, which served as a positive control.

Supplemental Experimental Procedures

Cell culture

All iPSCs were cultivated on irradiated mouse embryonic fibroblasts C3H (MEFs; kindly provided by T. Cantz, Hannover Medical School) in F12/DMEM medium (Gibco) supplemented with 20% knockout serum replacement (Gibco), 100 U/ml penicillin and 100 µg/ml streptomycin (PAA), 2 mM L-glutamine (Biochrom), 1% non-essential amino acids (Gibco), 0.1 mM β-mercaptoethanol (Sigma-Aldrich) and 20 ng/ml β-FGF (kindly provided by the Department of Technical Chemistry, Leibniz University Hannover). Colonies were picked once per week onto new MEFs in the presence of 10 µM Y-27632 (Tocris). MEFs were seeded in low-glucose DMEM (PanBiotech) supplemented with 15% FCS (Brazil One, PanBiotech), 100 U/ml penicillin and 100 µg/ml streptomycin, 2 mM L-glutamine, 1% non-essential amino acids and 0.1 mM β-mercaptoethanol onto coated plates using 0.1% bovine gelatin (Sigma Aldrich) for 30 min.

Plasmids

An all-in-one CRISPR/Cas9 plasmid was designed to perform gene editing. The Cas9 endonuclease derived from *Streptococcus pyogenes* was cloned behind a spleen focus-forming virus (SFFV) promoter followed by a T2A.dTomato cassette to identify transfected/transduced cells. The human U6 promoter was inserted to drive expression of the respective sgRNA. Two *BsmBI* recognition sites, which are separated by a spacer sequence, were cloned in front of the sgRNA scaffold. The orientation of the two BsmBI recognition sites allows an easy introduction of phosphorylated and annealed oligodeoxynucleotides to insert the 20 nt target sequence directly in frame in front of the sgRNA scaffold. Phosphorylation was accomplished for 45 min at 37°C with T4 polynucleotide kinase (NEB) using 100 pmol each of oligodeoxynucleotides 5'p47.ex2 and 3'p47.ex2 for sgRNA p47.ex2 and of oligodeoxynucleotides 5'p47.in1 and 3'p47.in1 for sgRNA p47.in1, respectively (Table S1). Annealing was performed by incubation at 98°C for 150 s followed by a cool down to 22°C at a rate of -0.1°C/min.

For gene correction, a donor construct (pMA.NCF1) was designed carrying the *NCF1* cDNA spanning exons 2 to 11, including the endogenous splice acceptor site in front of exon 2. Behind the polyadenylation signal of the *NCF1* cassette, a loxP-site flanked puromycin selection cassette was added driven by the phosphoglycerate kinase (PGK) promoter. The whole construct is flanked by two 700 bp homology arms, which were generated via PCR amplification from the genome of the parental iPSC clone using primers HAL_fw and HAL_rev or HAR_fw and HAR_rev, respectively (Table S1).

On-/off-target reporter cell assay

To evaluate the performance of our sgRNAs (p47.ex2 and p47.in1), we designed a dual-fluorescent reporter. Therefore, the 20 nt sgRNA target sequence plus the PAM motif for each on-target site (p47.ex2: ATGGCACCAGGAACATGTACACCTGGG, p47.in1: ATGGTCTACCAAAAAATGCGAGCTGGG) were amplified via PCR in frame with and in front of a superfolder green fluorescent protein (sfGFP) and cloned into a lentiviral backbone behind an SFFV promoter. An IRES.Puromycin selection cassette was inserted behind the sfGFP. For the off-target reporter, the respective sequence of the pseudogenes (p47.ex2: ATGGTTCACCAGGAACATGTACCTGGG, p47.in1: ATGGGTCTCTACCAAAAACGAGCTGGG) was amplified via PCR in frame with and in front of a superfolder enhanced blue fluorescent protein (sfBFP2) and cloned into the lentiviral backbone, followed by an IRES.Zeocin cassette for selection. Virus production was performed using a split packaging system as described before (Maetzig *et al.*, 2014). HT1080 cells were co-transduced in the presence of 4 µg/ml protamine sulfate (Sigma Aldrich) with both reporter constructs and selected via 5 µg/ml Puromycin and 100 µg/ml Zeocin. Upon transduction of the reporter cell line with the CRISPR/Cas9 vectors, sgRNA on-/off-target efficiency was assessed as loss of fluorescence using flow cytometry (LSRII, BD).

CRISPR/Cas9 ribonucleoprotein and AAV2 donor delivery

The AAV2 donor was generated by cloning the *NCF1* minigene cassette between the inverted terminal repeats of the plasmid pSUB201 (Samulski *et al.*, 1989) via blunt end ligation. Vector production and purification was performed as described elsewhere using the newly cloned AAV plasmid instead of pAAV/EGFP (Hacker *et al.*, 2005). Genomic titer of vector preparation was determined by qPCR using donor DNA specific primers (Puro_fw and Puro_rev). For RNP formation, 300 pmol sgRNA (Synthego) were incubated with 40 pmol Alt-R® S.p. Cas9 Nuclease V3 (Integrated DNA Technologies) for 10 min at room temperature in a total volume of 5 µl and nucleofected in $5x10^5$ p47-ΔGT iPSCs as described before. The cells were seeded on a Geltrex-coated 12-well plate, incubated for 20 min at 37°C and transduced with the AAV2 vector at a particle-per-cell ratio of 10,000.

Genetic analyses

DNA was isolated from iPSCs using the QIAamp DNA Blood Mini Kit (Qiagen). To analyze the overall gene editing frequency in nucleofected and sorted iPSCs, exon 2 of *NCF1* was amplified using the MyFi PCR mix (BioLine) with primers NGS_fw and NGS_rev. In a second PCR, each sample was amplified using distinct index primers as described elsewhere (Selich *et al.*, 2016). All PCR products were pooled and sequenced using ion torrent next generation sequencing. Deep sequencing results were screened for index primers to assign sequences to the different samples with a custom Perl 5 script (https://www.perl.org). In a second step, sequences were trimmed with the flanking parts "GGCTGAATG" and "CAGGACCTGT" embedding a region of interest. The alignment was performed with a custom R (https://www.R-project.org/) script, employing the packages Biostrings and msa (Pages *et al.*, 2018; Bodenhofer *et al.*, 2015).

To identify single p47-ΔGT iPSC clones, the Phire Hot Start II DNA Polymerase (Thermo Scientific) was used to amplify exon 2 using primers p47.2_fw and p47.2_rev (543 bp amplicon). PCR products were isolated from a 1% agarose gel (Invitrogen) and digested for 30 min with the restriction enzyme BsrGI (NEB). The wild type *NCF1* sequence was cleaved by BsrGI, which resulted in 362 bp and 181 bp fragments. The GT-deletion caused a loss of the BsrGI recognition site; thus, the lack of cleavage products indicated loss of the wild type sequence. Positively identified clones were confirmed by Sanger sequencing (Seqlab).

To verify genetically corrected clones, all PCRs were performed using the Phusion Green Hot Start II High-Fidelity PCR Master Mix (Thermo Scientific). To detect the 5'junction of the inserted donor construct primers 1F and 1R were used (850 bp amplicon). The 3'junction was amplified using primers 2F and 2R (801 bp amplicon). Random insertion of the plasmid was addressed by amplifying the 5' and 3' ends of the plasmid with primers 3F/1R (1148 bp amplicon) and 4F/3R (1232 bp amplicon), respectively. All PCR products were analyzed by 1% agarose gel electrophoresis.

Intact pseudogene sequences were analyzed by amplification of wild type sequences around the Cas9 cleavage site in a nested PCR using primers W1F and W1R (481 bp amplicon) and W2F and W2R (194 bp amplicon) and the Phusion Green Hot Start II High-Fidelity PCR Master Mix. After Sanger sequencing of the extracted PCR fragments, sequences were identified based on the presence or absence of specific indels and assigned to *NCF1*, *NCF1B* or *NCF1C*. All primers are listed in Table S1.

Minigene copy number determination by quantitative PCR (qPCR)

Genomic DNA was isolated from corrected clones using the QIAamp DNA Blood Mini Kit. Primers specific for the puromycin resistance gene (Puro_fw and Puro_rev) were used to determine the minigene copy number and are described in Table S1. The qPCR was performed in triplicates with the Step One Plus Real Time PCR System (Applied Biosystems, Life Technologies) and the QuantiTect SYBR Green PCR Kit (Qiagen) according to the manufacturer's manual. The copy number of the puromycin resistance gene was normalized to *PTBP2* levels.

Generation of patient-derived p47^{phox}-deficient (p47-CGD) iPSCs

The human blood sample was collected after written informed consent of the donor at Hannover Medical School (Germany). Peripheral blood mononuclear cells (PBMCs) were isolated from 9 ml peripheral blood using a ficoll gradient (Biochrom). Cells were reprogrammed into iPSCs as described elsewhere (Warlich *et al.*, 2011). Briefly, 2×10^5 cells were transduced with a lentiviral vector expressing the four transcription factors OCT4, KLF4, SOX2 and c-MYC in the presence of 8 µg/ml polybrene (Sigma Aldrich). The cells were cultured in HSC medium consisting of StemSpan (STEMCELL Technologies) supplemented with 100 U/ml penicillin and 100 µg/ml streptomycin, 100 ng/ml stem cell factor, 50 ng/ml thrombopoietin and 100 ng/ml fms-like tyrosine kinase 3 (all cytokines: Peprotech). The day after transduction, 50 µg/ml 2-phospho-L-ascorbic acid (Sigma-Aldrich) and 2 mM valproic acid (Ergenyl®, Sanofi-Aventis) were added to the medium. Three days after transduction, cells were transferred onto MEFs and cultured in HSC and F12/DMEM-iPSC medium in a mixture of 1:1. Medium was changed twice a week using F12/DMEM-iPSC medium. After three to four weeks, one iPSC colony formed and was analyzed for pluripotency.

Pluripotency assays

All iPSCs were analyzed for TRA-1-60 and SSEA-4 expression using flow cytometry. Therefore, iPSCs were trypsinized, resuspended into single cells, blocked with human FcR block (Miltenyi) for 10 min and stained with TRA-1-60-PE (Miltenyi) and SSEA-4-Alexa Fluor 647 (BD) for 30 min at 4°C. Data was acquired using a CytoFlex S flow cytometer (Beckman Coulter).

Expression levels of the pluripotency markers *NANOG*, *OCT4* and *DNMT3B* were analyzed via qPCR. Therefore, RNA was isolated from each iPSC clone using the RNeasy Mini Kit (Qiagen) and reverse transcribed into cDNA using the QuantiTect Reverse Transcription Kit (Qiagen) as per manufacturer's instruction. The qPCR was

performed in triplicates with the Step One Plus Real Time PCR System and the QuantiTect SYBR Green PCR Kit according to the manufacturer's manual. Primer sequences for *NANOG*, *OCT4*, *DNMT3B* and *β-ACTIN* are described in Table S1. The expression level of each target gene was normalized to *β-ACTIN* expression levels and expressed in relation to the embryonic stem cell (ESC) clone H9 using the ΔΔCt method (Pfaffl, 2001).
The potential of generated iPSCs to differentiate into cells of all three germ layers was assessed in a teratoma formation assay as described before (Philipp *et al.,* 2018). Briefly, 3×10^6 iPSCs were resuspended in 100 µl F12/DMEM-iPSC medium containing 20 µM Y-27632. After addition of 100 µl Matrigel basement membrane matrix (Corning), the cell suspension was subcutaneously injected into the flanks of an NBSGW mouse (NOD.Cg-Kit^{W-41J} Tyr^{+} $Prkdc^{scid}$ $Il2rg^{tm1Wjl}$/ThomJ). The animal experiment was performed in accordance with Lower Saxony State Office for Consumer Protection and Food Safety in Germany. The mouse was sacrificed after the teratoma reached a diameter of 1.5 cm. After isolation and fixation of the teratoma in 4% buffered formaldehyde (Carl Roth), the tissue was embedded in paraffin, sectioned into 3 µm slices and stained with hematoxylin and eosin. Microscopic analysis was performed with an Olympus BX51 microscope (software Cell^F version 3.4, Olympus).

Myeloid differentiation of iPSCs
Differentiation of iPSCs into granulocytes and macrophages was performed using an embryoid body (EB)-based protocol as previously described (Lachmann *et al.*, 2015). Briefly, iPSC colonies were detached using dispase (Roche) and transferred into EB medium (KO/DMEM medium (Gibco) supplemented with 20% knockout serum replacement (Gibco), 100 U/ml penicillin and 100 µg/ml streptomycin (PAA), 2 mM L-glutamine (Biochrom), 1% non-essential amino acids (Gibco), 0.1 mM β-mercaptoethanol (Sigma-Aldrich) and 10 µM Y-27632 (Tocris)). EB formation was done for 5 days on an orbital shaker at 80 rpm. For granulocyte differentiation, EBs were picked into APEL2 medium (StemCell Technologies) supplemented with 100 U/ml penicillin and 100 µg/ml streptomycin, 50 ng/ml human granulocyte colony-stimulating factor (G-CSF, Peprotech) and 25 ng/ml human interleukin-3 (Peprotech). For macrophage differentiation, EBs were transferred into X-Vivo15 medium (Lonza) supplemented with 100 U/ml penicillin and 100 µg/ml streptomycin, 2 mM L-glutamine, 50 ng/ml human macrophage colony-stimulating factor (M-CSF, Peprotech) and 25 ng/ml human interleukin-3. Differentiated cells were harvested once per week and cultured in RPMI 1640 medium (PanBiotech) supplemented with 10% fetal calf serum (FCS, Brazil One, PanBiotech), 100 U/ml penicillin and 100 µg/ml streptomycin, 2 mM L-glutamine, 100 ng/ml G-CSF or M-CSF, respectively.

Intracellular p47^{phox} staining
The FoxP3 intracellular staining kit (Biolegend) was used for fixation and permeabilization of differentiated granulocytes. Briefly, cells were fixed for 20 min in Fix/Perm solution, washed once with PBS and once with Perm buffer. After incubation in Perm buffer for 15 min, cells were stained with the primary p47^{phox} antibody (BD) for 30 min. Secondary antibody staining was performed for 30 min using a goat-anti-mouse DyLight488 antibody (Thermo Fisher). Stained cells were analyzed using a CytoFlex S flow cytometer.

Pappenheim staining
To analyze cell morphology, 5×10^4 cells were spun onto microscope glass slides using a Cytospin 4 (Thermo Scientific), stained with May-Grünwald and Giemsa according to manufacturer's instructions (Pappenheim staining, Sigma-Aldrich) and analyzed with an Olympus BX51 microscope (software Cell^F version 3.4, Olympus).

Functional granulocyte assays
The dihydrorhodamine (DHR) assay was performed as described elsewhere (Dreyer *et al.*, 2015), with minor modifications. Briefly, 10^5 cells were resuspended in 500 µl Hank's Buffered Salt Solution (HBSS) containing Mg/Ca (Gibco). After stimulation with 40 nM phorbol 12-myristate 13-acetate (PMA; Sigma Aldrich) in the presence of 1000 U catalase (Sigma Aldrich) for 5 min, 250 ng DHR (Sigma Aldrich) were added for 15 min to the cells. Cells were placed on ice to stop the reaction and analyzed by flow cytometry (CytoFlex S).
ROS production was assessed using a luminol chemiluminescence assay. Therefore, 10^5 cells were resuspended in 200 µl HBSS containing Mg/Ca, supplemented with 0.5% human albumin (Grifols) and 0.5 mM luminol (Sigma Aldrich). After stimulation with 40 nM PMA, ROS production was measured every 2 min for 60 min at 37°C at a multi-mode plate reader (Beckman Coulter).
NET formation was quantified as described before (Dreyer *et al.*, 2015), with minor modifications. For each sample, 3.5×10^4 cells were stimulated with 40 nM PMA and stained with 1 mM Sytox Green (Invitrogen) to visualize the DNA. NET formation was quantified using a fluorescence microplate reader (Berthold technologies) with a filter setting of 485/535 nm (excitation/emission).

Bacteria killing assay

Competent XL-1 blue *E. coli* bacteria were transformed with a plasmid carrying sfGFP, plated on LB-agar plates (CarlRoth) supplemented with 75 µg/ml ampicillin (Ratiopharm) and grown overnight. The next day, 10^5 macrophages were seeded in a 48-well plate in antibiotic-free medium (RPMI 1640 (PanBiotech) supplemented with 10% FCS, 2 mM L-glutamine and 100 ng/ml M-CSF). An sfGFP-positive colony was picked, transferred into 100 ml LB-medium (CarlRoth) plus ampicillin and grown to an OD_{600} of 0.5 overnight (~12-14 h). Macrophages were infected with the bacteria at an MOI of 1, and 40 µg/ml meropenem (Hexal) was added to the medium one hour post-infection. After another 24 h, all cells were harvested and lysed in ultrapure water (Biochrom) for 2 min. Serial dilutions were made in PBS (Lonza), plated on LB-agar plates supplemented with ampicillin and incubated overnight to determine the number of colony forming units. Prior to cell lysis, fluorescence microscope pictures were taken using a Zeiss Observer Z1 microscope (AxioVision software). Additional wells with macrophages were infected to measure the phagocytosis rate by flow cytometry (FACS Calibur).

Statistical tests

Statistical significance was determined using one-way or two-way ANOVA with GraphPad Prism 7. All bar graphs represent the mean ± SD. *P≤0.05, **P≤0.01, ***P≤0.001, ****P≤0.0001.

Table S1: Primers/oligodeoxynucleotides used in this study. Related to Figure 1, Figure 2, Figure S2, Figure S3 and Figure S4.

Name	Sequence (5'-3')
p47.ex2 donor (ssODN)	GAACTCGTAGATCTCGGTGAAGCGCCGGTAGACCACCTTCTCCGACAGGTCCTGC CATTTCACCAGGAACATGTACCTGGAGGAAAGCCAGAGTCGGGGGACCCCATTCA GCCTCCAAAG
5'p47.ex2	CACCGCACCAGGAACATGTACACCT
3'p47.ex2	AAACAGGTGTACATGTTCCTGGTGC
5'p47.in1	CACCGTCTACCAAAAAATGCGAGCT
3'p47.in1	AAACAGCTCGCATTTTTTGGTAGAC
HAL_fw	AGCGCTATAGATCTTGTGGAGATGAGGTTTCACT
HAL_rev	AGAGTCGGGCTAGCCATGGTGGCGCGCACCTGTA
HAR_fw	TTCTGCAGACGCGTGACAGGGTTTTGCCATATTG
HAR_rev	CTAGAGTCGCGGCCGCGCCTGACCAATATGGTGAAA
NGS_fw	GGTGGGTTTTCCAGTCACACGCCCTTTCTGCAATCCAGGACAACC
NGS_rev	TTCGTTGGGAGTGAATTAGCCTGGGTCCCTGTCCCTCCTCCA
p47.2_fw	CTGGGGCGTGGCAGCACTTGGGT
p47.2_rev	GCTATGATTGCGCCCCTGCACTGCAA
1F	ACACCACCATGCCTGGCTAGTT
1R	TAAGGTTTTATGGAACTCGTAGATCTC
2F	TACGAAGTTATTCTGCAGACGCG
2R	GTTGGCTCCCGTCTGTAATCC
3F	TAACCAATAGGCCGAAATCG
4F	GCATCGCATTGTCTGAGTAGG
3R	TATAGTCCTGTCGGGTTTCG
W1F	CCGATTATCCTGCTTGTCCTCTGCAGTG
W1R	ATGGTGGCGCATGCCTGTAATCC
W2F	AGAGATTCTCCTGCCTCAGCC
W2R	ATGCAGTGAGTGGAGATTGTGCC
NANOG_fw	TCACACGGAGACTGTCTCTC
NANOG_rev	GAACACAGTTCTGGTCTTCTG
OCT4_fw	CCTCACTTCACTGCACTGTA
OCT4_rev	CAGGTTTTCTTTCCCTAGCT
DNMT3B_fw	ATAAGTCGAAGGTGCGTCGT
DNMT3B_rev	GGCAACATCTGAAGCCATTT
β-ACTIN_fw	TACCTGTATAGTGTACTTCAT
β-ACTIN_rev	GGTCATGAGAAGTGTTGCTA
Puro_fw	CACCAGGGCAAGGGTCTG
Puro_rev	CTCGTAGAAGGGGAGGTTGC
PTBP2_fw	TCTCCATTCCCTATGTTCATGC
PTBP2_rev	GTTCCCGCAGAATGGTGAGGTG

Supplemental References

Bodenhofer U, Bonatesta E, Horejs-Kainrath C, Hochreiter S (2015). "msa: an R package for multiple sequence alignment." Bioinformatics, 31(24), 3997–3999. doi: 10.1093/bioinformatics/btv494.

Dreyer, A., Hoffmann, D., Lachmann, N., Ackermann, M., Steinemann, D., Timm, B., Siler, U., Reichenbach, J., Grez, M., Moritz, T. et al. (2015). TALEN-mediated functional correction of X-linked chronic granulomatous disease in patient-derived induced pluripotent stem cells. Biomaterials *69*, 191-200.

Hacker, U.T., Wingenfeld, L., Kofler, D.M., Schuhmann, N.K., Lutz, S., Herold, T., King, S.B.S., Gerner, F.M., Perabo, L., Rabinowitz, J. et al. (2005). Adeno-associated virus serotypes 1 to 5 mediated tumor cell directed gene transfer and improvement of transduction efficiency. J Gene Med *7*, 1429-1438.

Lachmann, N., Ackermann, M., Frenzel, E., Liebhaber, S., Brennig, S., Happle, C., Hoffmann, D., Klimenkova, O., Lüttge, D., Buchegger, T. et al. (2015). Large-scale hematopoietic differentiation of human induced pluripotent stem cells provides granulocytes or macrophages for cell replacement therapies. Stem Cell Reports 4, 282-296.

Maetzig, T., Kuehle, J., Schwarzer, A., Turan, S., Rothe, M., Chaturvedi, A., Morgan, M., Ha, T.C., Heuser, M., Hammerschmidt, W. et al. (2014). All-in-One inducible lentiviral vector systems based on drug controlled FLP recombinase. Biomaterials *35*, 4345-4356.

Pagès H, Aboyoun P, Gentleman R, DebRoy S (2018). Biostrings: Efficient manipulation of biological strings. R package version 2.50.1.

Pfaffl, M.W. (2001). A new mathematical model for relative quantification in real-time RT-PCR. Nucleic Acids Res. 29, 45e–45.

Philipp, F., Selich, A., Rothe, M., Hoffmann, D., Rittinghausen, S., Morgan, M.A., Klatt, D., Glage, S., Lienenklaus, S., Neuhaus, V. et al. (2018). Human Teratoma-Derived Hematopoiesis Is a Highly Polyclonal Process Supported by Human Umbilical Vein Endothelial Cells. Stem Cell Reports *11*, 1051-1060.

Samulski R.J., Chang L.S., Shenk T. (1989). Helper-Free Stocks of Recombinant Adeno-Associated Viruses: Normal Integration Does Not Require Viral Gene Expression. J Virol *63*, 3822-3828.

Selich, A., Daudert, J., Hass, R., Philipp, F., von Kaisenberg, C., Paul, G., Cornils, K., Fehse, B., Rittinghausen, S., Schambach, A. et al. (2016). Massive Clonal Selection and Transiently Contributing Clones During Expansion of Mesenchymal Stem Cell Cultures Revealed by Lentiviral RGB-Barcode Technology. Stem Cells Transl Med *5*, 591-601.

Warlich, E., Kuehle, J., Cantz, T., Brugman, M.H., Maetzig, T., Galla, M., Filipczyk, A.A., Halle, S., Klump, H., Schöler, H.R. et al. (2011). Lentiviral vector design and imaging approaches to visualize the early stages of cellular reprogramming. Mol. Ther. *19*, 782-789.
